# Discovery of New Ginsenol-Like Compounds with High Antiviral Activity

**DOI:** 10.3390/molecules26226794

**Published:** 2021-11-10

**Authors:** Aleksandrina S. Volobueva, Olga I. Yarovaya, Marina V. Kireeva, Sophia S. Borisevich, Kseniya S. Kovaleva, Iliya Ya. Mainagashev, Yuri V. Gatilov, Margarita G. Ilyina, Vladimir V. Zarubaev, Nariman F. Salakhutdinov

**Affiliations:** 1 Laboratory of Experimental Virology Pasteur Institute of Epidemiology and Microbiology, 14 Mira Str., 197101 St. Petersburg, Russia; sasha-khrupina@mail.ru (A.S.V.); zarubaev@gmail.com (V.V.Z.); 2 Department of Medicinal Chemistry, N. N. Vorozhtsov Novosibirsk Institute of Organic Chemistry, Siberian Branch of the Russian Academy of Sciences, Lavrentiev Ave. 9, 630090 Novosibirsk, Russia; kseniya.kovaleva3103@yandex.ru (K.S.K.); ilmai@nioch.nsc.ru (I.Y.M.); gatilov@nioch.nsc.ru (Y.V.G.); anvar@nioch.nsc.ru (N.F.S.); 3 Biology Department, St. Petersburg State University, 7/9 Universitetskaya Nab., 199034 St. Petersburg, Russia; pressa@spbu.ru; 4 Laboratory of Chemical Physics, Ufa Institute of Chemistry Ufa Federal Research Center, 71 Pr. Oktyabrya, 450078 Ufa, Russia; monrel@mail.ru (S.S.B.); margarita.kondrova@yandex.ru (M.G.I.)

**Keywords:** Ginsenol, Ginsamide, antiviral agent, surface protein, influenza, resistance, molecular modeling

## Abstract

A number of framework amides with a ginsenol backbone have been synthesized using the Ritter reaction. We named the acetamide as Ginsamide. A method was developed for the synthesis of the corresponding amine and thioacetamide. The new compounds revealed a high activity against H1N1 influenza, which was confirmed using an animal model. Biological experiments were performed to determine the mechanism of action of the new agents, a ginsamide-resistant strain of influenza virus was obtained, and the pathogenicity of the resistant strain and the control strain was studied. It was shown that the emergence of resistance to Ginsamide was accompanied by a reduction in the pathogenicity of the influenza virus.

## 1. Introduction

Influenza is a highly contagious human disease. Every year, 500 million people on the planet contract influenza, and around 2 million die. The influenza virus causes annual epidemics, as well as pandemics when a new antigenic version of the virus becomes available, affecting all regions of the world, with high morbidity, hospitalisation, and mortality in all age groups. Pandemic influenza A(H1N1)pdm09 had widespread coverage in many countries, a severe clinical course, and high mortality [1]. Adverse outcomes were most often observed not only in individuals with comorbid chronic disease [2] but also in young people without significant previous pathology, including pregnant women [3]. Human infection with avian influenza viruses of subtypes H5N1, H7N7, and H7N9 has also been observed in recent years [4].

Medicines with different mechanisms of activity are now available for the prevention and treatment of influenza infection. These are primarily etiotropic drugs that target specific viral targets. Two groups of chemicals, adamantane derivatives (amantadine and its Russian equivalent, remantadine) [5] and the viral neuraminidase inhibitors oseltamivir (Tamiflu^®^) and zanamivir (Relenza^®^), are internationally recognized as anti-influenza medicines [6]. In the United States, two other neuraminidase inhibitors, intravenous peramivir (Rapiact^®^) [7] and inhaled laninamivir (Inavir^®^), have received FDA approval [8]. Neither peramivir nor laninamivir are certified in Russia. In 2018, a new drug baloxavir marboxil (Xofluza^®^), which blocks viral endonuclease activity, was registered in the United States [9].

**Figure 1:**
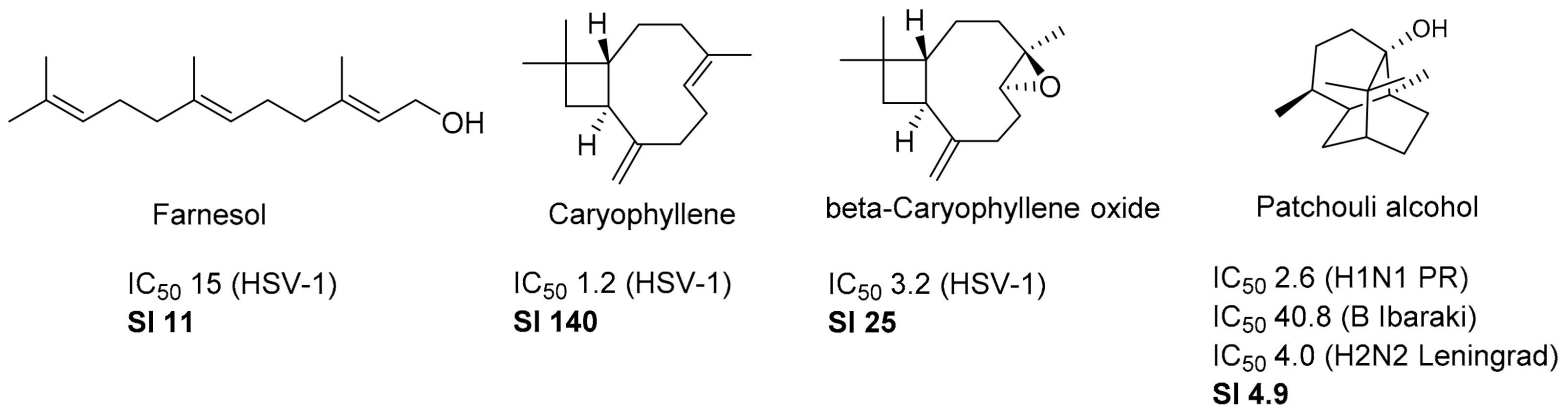
Structures of sesquiterpenoids with antiviral activity.

Natural compounds and their synthetic derivatives are extremely important components of effective drugs [10,11]. They are of great importance for the development of the world’s pharmaceutical industry since they currently constitute a significant part of all known medical drugs [12,13]. Among the commercially available natural compounds, substances of the mono- and sesquiterpene series deserve special attention [14,15]. Since ancient times, man has used essential oils of spicy and aromatic plants to treat diseases of the upper respiratory tract. For a long time, the basis of folk medicines were water and water-alcoholic extracts of medicinal herbs and other plant raw materials, despite the fact that their active components were not yet known. Currently, available compounds of the terpene series are a convenient source of initial building blocks in the synthesis of effective antiviral agents [16,17]. Thus, our team of researchers previously discovered a new class of antiviral agents that are active against the influenza virus—(+)-camphor iminoderivatives [18]. Among the substances described by us, Camphecene imino-alcohol—a product of the interaction between camphor and aminoethanol—showed the greatest activity against a wide range of influenza viruses [19]. Meanwhile, borneol ester derivatives showed high activity against the filoviruses Marburg and Ebola [20]; camphene-based ethers showed a wide range of antiviral activity [21]; camphor-based hydrazones were found to be active against variolaviruses, including the smallpox virus [22]; and camphor and fenchone conjugates showed high activity against Hantaan viruses [23]. At the same time, less attention has been devoted to synthetic transformations of natural compounds belonging to the sesquiterpene class. This is primarily due to the high commercial cost of sesquiterpene compounds. Nevertheless, three of the best-known sesquiterpenoids—the acyclic alcohol farnesol, the bicyclic natural cycloalkene caryophyllene and its 4β,5α-epoxy derivative—have been described as having antiviral activity against HSV-1 virus [24] (Figure 1). The authors of the above article compared the antiviral activity of the above natural compounds and anise oil. Caryophyllene turned out to be the most active against HSV-1. The tricyclic carcass sesquiterpenoid patchouli alcohol has been shown to have activity against the influenza virus A/PR/8/34 (H1N1), influenza virus type B/Ibaraki/2/85 [25], and against the strain of influenza virus A/Leningrad/134/17/1957 (H2N2) [26].

Caryophyllene is the most widespread representative of a number of bicyclic sesquiterpenes and is present in many essential oils, especially the oils of *Eugenia caryophyllata* [27], *Myrica gale* [28], and *Comptonia peregrina* [29], which have a high content of caryophyllene. As a rule, isocaryophyllene ((*Z*)-β-caryophyllene), humulene, and the epoxy derivative β-caryophyllene oxide are associated with caryophyllene in essential oils [30]. In industry, this compound is isolated from clove oil waste. Many publications are devoted to the study of the biological activity of caryophyllene. Thus, it has been shown that this sesquiterpene is a selective agonist of cannabinoid receptor type 2 (CB2) [31] and exhibits pronounced analgesic activity [32], anticancer activity [33,34], antibacterial and antifungal activity [35]. A significant number of works are devoted to acid-catalysed reactions of caryophyllene and its epoxy derivatives. It has been shown that the main products under acidic conditions are compounds with caryolanic and clovanic backbones [36]. We previously studied the transformations of caryophyllene under Ritter reaction conditions and showed that the main product is a tricyclic acetamide with a caryolanic type of backbone [37].

In the present work, we synthesized compounds based on isocaryophyllene and studied the activity of the new substances against influenza viruses. For the most active substance, biological tests were performed to identify the mechanism of action, a resistant strain was obtained, and antiviral activity was studied using an animal model.

## 2. Results and Discussion

### 2.1. Chemistry

**Scheme 1:**
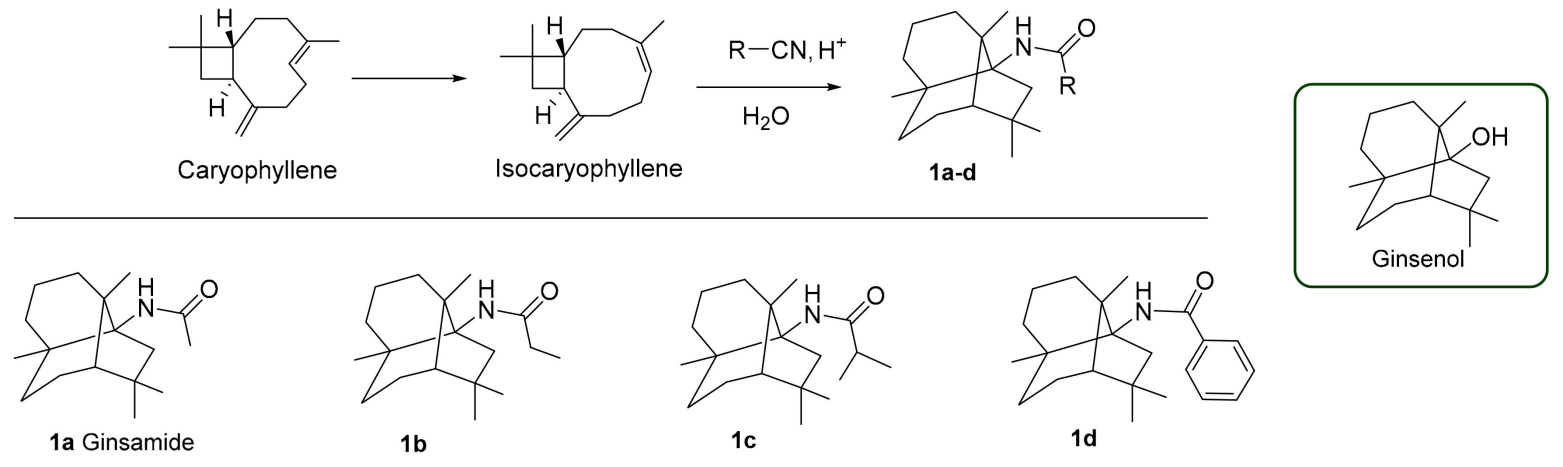
Synthetic pathway to target derivatives **1a**–**d**.

Amide bond formation is one of the most important trends in organic chemistry since this type of bond exists in many natural molecules—peptides, proteins, polymeric materials, and alkaloids. Due to the high importance of this bond, there are many methodologies for creating an amide bond. Among the widely known methods, the Ritter reaction, a method for the synthesis of N-substituted amides of carboxylic acids by alkylation of nitriles with carbocations, occupies a special position [38]. Under acid catalysis, alkenes or their derivatives, alcohols, oxiranes, etc., can act as carbocation precursors [39]. This reaction has attracted the attention of researchers due to the simplicity and availability of the reagents used, and the class of catalysts used in this reaction has recently been significantly expanded [40]. As noted in the introduction, we previously studied the behaviour of caryophyllene, isocaryophyllene, and their epoxy derivatives under Ritter reaction conditions [37]. The transformation of isocaryophyllene in acetonitrile, with the addition of 5% sulfuric acid followed by neutralization, led to the formation of tricyclic amide **1a**. The backbone of this compound coincides with the backbone of the natural sesquiterpene alcohol ginsenol, previously isolated from ginseng root [41]. Caryophyllene is a commercially available sesquiterpene; we have successfully scaled up the synthesis of its isocaryophyllene isomer by the previously described method [42]. In this work, we performed new transformations of isocaryophyllene with nitriles of various structures and showed that the formation of framed **1a**–**d** amides with acceptable yields occurs in all cases (Scheme 1). The reaction was accompanied by the formation of isocaryophyllene isomerization products, the structure of which we have not established in this work.

Compound **1a**, which we named Ginsamide, precipitated as crystals from acetonitrile after-treatment of the reaction mixture with an aqueous soda solution, and further recrystallization from a chloroform-hexane mixture led to a pure product. Purification of **1b–d** amides was carried out using column chromatography; the yield of pure products was from 32% to 68%.

**Figure 2:**
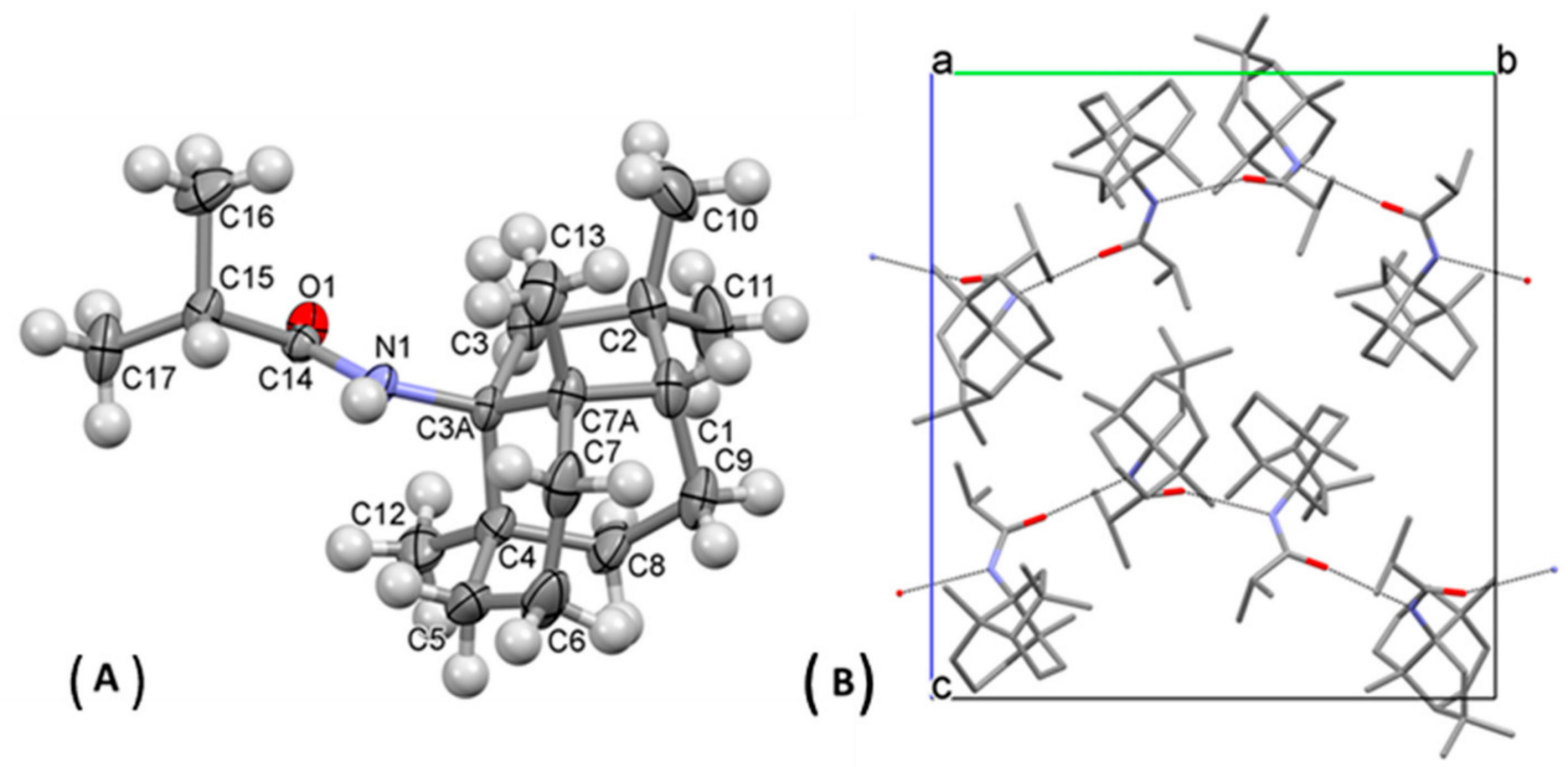
(**A**) Crystal structure of compound 1c (CCDC 2114089); (**B**) Molecules packing of 1c (view along a axis). Hydrogen atoms are not shown. Chains of molecules along the b axis are formed due to N-H…O H-bonds (H…O 2.20, 2.21 Å, N-H…O 168, 171°).

The crystal structure of the ginsamide **1a** was described earlier. In this work, we were able to obtain a crystal of compound **1c** that was suitable for an X-ray diffraction study. Figure 2 shows the spatial structure of this substance.

**Scheme 2:**
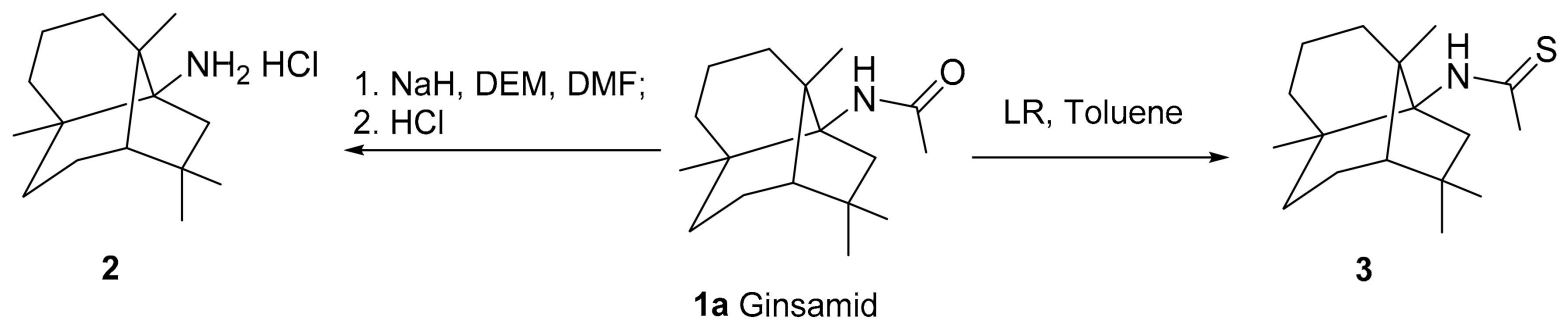
Synthetic pathway to target derivatives **2**–**3.** DEM: Diethyl malonate; LR: Lawesson’s reagent.

Since we isolated compound **1a** with good yields, we further set ourselves the goal of synthesizing analogues of this agent. In order to expand the synthetic possibilities of using the mentioned tricyclic amide, we tried to obtain a framework amine containing a ginsenol backbone and a primary amino group. The approach to ginsamine synthesis involves the removal of the acetyl group. However, this proved to be a nontrivial task. Acidic and basic hydrolysis of the amide part of ginsamide was attempted, namely, boiling the original amide with hydrochloric acid in various solvents, boiling the original with potassium hydroxide in pyridine, and heating the original with potassium hydroxide at 150 °C Further attempts were made to reduce with lithium aluminium hydride in tetrahydrofuran and using reduction with hydrogen in the presence of palladium on coal in an autoclave under pressure. The next approach was a nucleophilic substitution reaction with stronger nucleophiles. The use of thiourea and sodium hydrosulphide failed. Switching to a malone ether dianion as a nucleophilic agent led to the expected amine **2**. Boiling of the initial ginsamide **1a** with an excess of malone ether and sodium hydride in dimethylformamide at 135 °C for 6–8 h removed the acetyl group in an acceptable yield (about 60%, Scheme 2). Thus, for the first time, we have developed the synthesis of an amine-containing framework structure **2** that has not been previously described. In addition, we obtained thioacetamide **3** by treating compound **1a** with Lawesson’s reagent.

As a result of the chemical transformations performed, for the first time, we have obtained a number of amides having a framework fragment coinciding with the backbone of the natural sesquiterpene alcohol ginsenol, developed a method for the synthesis of the tricyclic amine, and obtained sulphur derivatives of ginsamide. All substances synthesized in this work were further investigated as influenza virus inhibitors.

### 2.2. Antiviral Activity Study

Influenza viruses are divided into three types: influenza A, B, C viruses; in particular, influenza A viruses have a long history of emergence and re-emergence. Therefore, the compounds obtained have been studied for antiviral activity against the influenza virus A/Puerto Rico/8/34 (H1N1) in MDCK cell cultures (Table 1). Oseltamivir, ribavirin, and rimantadine were used as reference compounds.

All of the **1a**–**d** amides synthesized using the Ritter reaction showed activity against influenza virus H1N1. The amides containing aliphatic fragments were the most promising for further study because they showed activity in the submicromolar range. Among the agents **1a**–**c**, substance **1b** was more toxic on the studied cell line. Amine **2** showed activity in the lower micromolar range but had a rather high toxicity. The thio-substituted agent **3** was significantly less toxic than amine **2**. Based on the results of analysis of the small library, one can conclude that compound **1a** possesses the highest virus-inhibiting activity (lowest IC_50_, 0.152 μM) whilst having the lowest toxicity (CC_50_ > 1140 μM, SI = 7500). We, therefore, focused our further investigations on this leading compound, hereafter referred to as Ginsamide (GS).

To confirm the antiviral activity of the lead compound, we performed a virus yield reduction test. Similar to the cytopathic effect CPE) reduction assay, the compound demonstrated strong dose-dependent virus inhibition against influenza virus A/Puerto Rico/8/1934 (H1N1), with IC_50_ = 0.15 μM. Given that its CC_50_ > 1140 μM, its SI was calculated as 7500, which is identical to the SI calculated previously, based on the results of the CPE reduction assay. Ginsamide, therefore, possesses direct antiviral activity.

**Figure 3:**
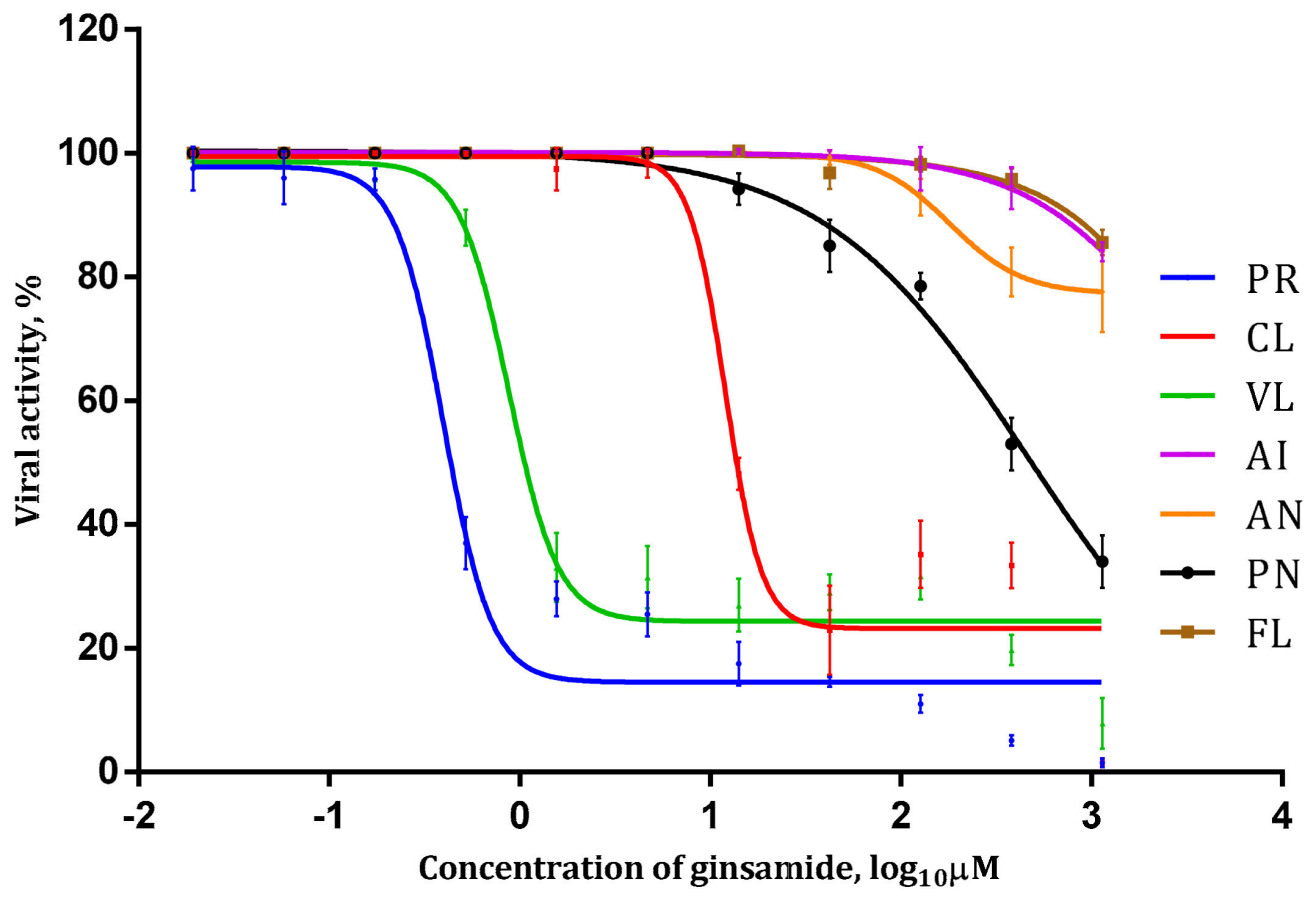
Virus-inhibiting activity of ginsamide against influenza viruses based on cytoprotection assay results. MDCK cells were infected with different influenza viruses (MOI 0.01) in the presence of serial dilutions of ginsamide. After incubation for 72 h, cell viability was evaluated by the MTT test. Virus designation: PR: A/Puerto Rico/8/34 (H1N1); CL: A/California/07/09 (H1N1)pdm09; VL: A/Vladivostok/2/2009 (H1N1); AI: A/Aichi/2/68 (H3N2); AN: A/Anhui/1/2013 (H7N9); PN: A/mallard/Pennsylvania/10218/84 (H5N2); FL: B/Florida/4/2006.

In order to assess whether the anti-influenza activity of ginsamide is subtype-specific or has a wide spectrum of activity, we repeated the cytoprotection test using influenza viruses of other types and subtypes (Table 2, Figure 3). The results demonstrated that the virus-inhibiting activity of ginsamide is strongly subtype-specific. The highest activity was observed for influenza viruses of H1 and H1pdm09 subtypes, while the values of IC_50_ appeared one to three orders of magnitude higher against other viruses. Importantly, some of the viruses used in the study are amantadine-resistant, and A/Vladivostok/2/2009 (H1N1) virus is resistant to oseltamivir. The high inhibiting activity of GS to these viruses suggests that its target differs from the traditionally used M2 ion channel and neuraminidase utilized by amantadine/rimantadine and oseltamivir, respectively.

### 2.3. A Study of the Mechanism of Antiviral Activity

**Figure 4:**
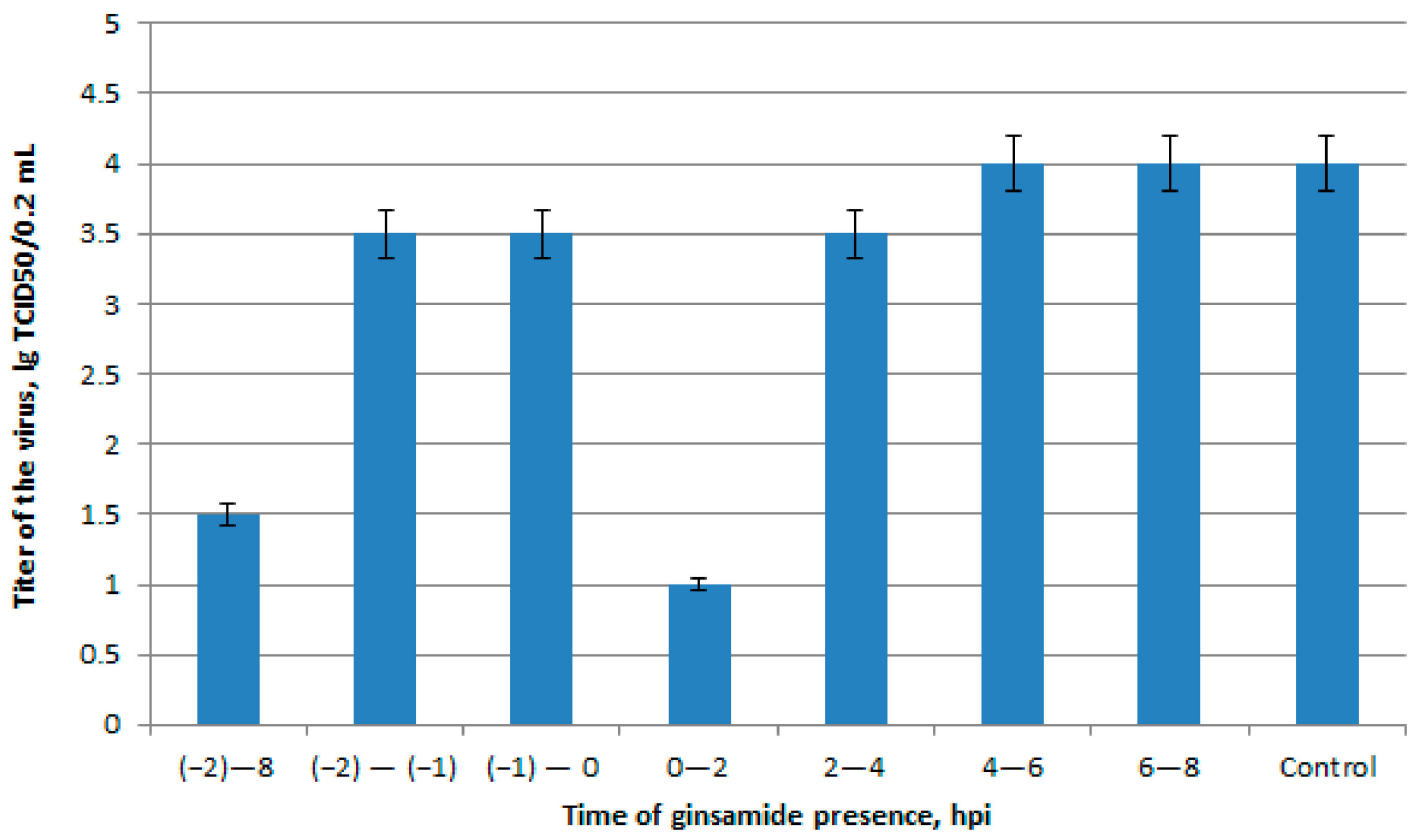
Activity of ginsamide (GS) against influenza virus A/Puerto Rico/8/34 (H1N1) according to the time-of-addition experiment. MDCK cells were infected with influenza virus, and GS (200 μM) was added at the indicated time points, where 0 corresponds to the moment when the cells were infected. The infectious activity of the viral progeny was tested by further titration in the MDCK cells.

To assess the mechanism of the antiviral activity of ginsamide, we focused on what stage of the viral cycle demonstrated the highest activity. Based on these results, it would be possible to deduce the viral or cellular proteins that could be a target for the compound. For this purpose, we performed a time-of-addition assay. We cultivated influenza virus A/Puerto Rico/8/34, adding and removing ginsamide at different time points regarding the infecting time. After one cycle of reproduction, the infectious titre of viral progeny was determined by TCID_50_ assay (Figure 4).

As can be seen from the data presented, the most pronounced effect of ginsamide was reached when it was present in the culture medium from 0 to 2 h post-infection (hpi). This period corresponds to the virus’ attachment to the cell surface, endocytosis, endosome acidification, and the fusion of viral and cellular membranes.

Thus, we have shown that ginsamide demonstrates the highest efficacy when added to infected cells at the early stages of the viral cycle.

**Figure 5:**
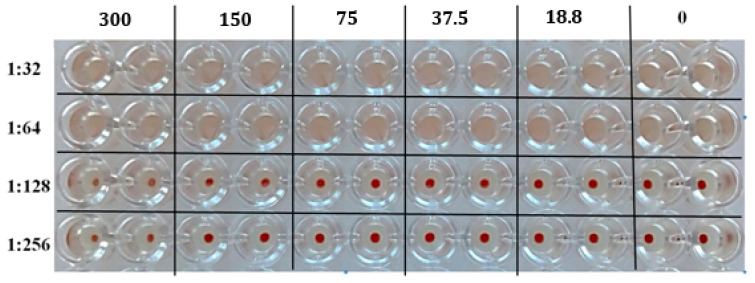
Effect of ginsamide on influenza virus-induced hemagglutination. Two-fold dilutions of influenza virus A/Puerto Rico/8/34 (H1N1) (indicated on the left) were incubated with ginsamide at a range of concentrations (0–300 µg/mL) in the wells of round-bottom plates, and 1% chicken erythrocytes were added. The plates were incubated for 1 h at 20 °C for sedimentation of erythrocytes.

In addition, to assess the impact of ginsamide on the functions of viral hemagglutinin (HA), we performed two direct assays to test its receptor-binding and fusogenic activity in the presence of GS. It was shown that GS did not change the hemagglutinating titre of the virus (Figure 5). GS, therefore, does not affect the receptor-binding activity of HA. No other compound from the library studied has demonstrated fusion inhibiting activity either (data not shown).

**Figure 6:**
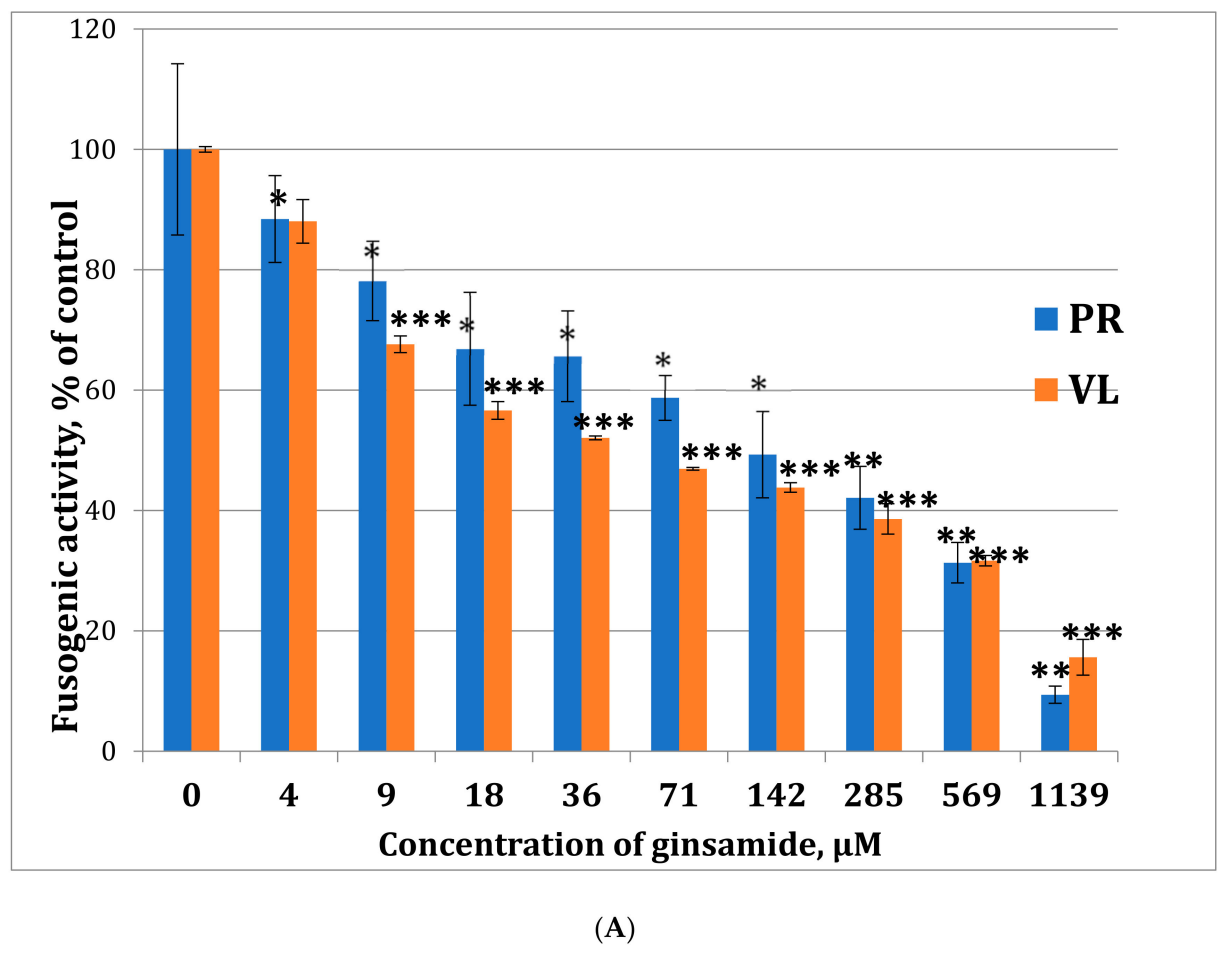

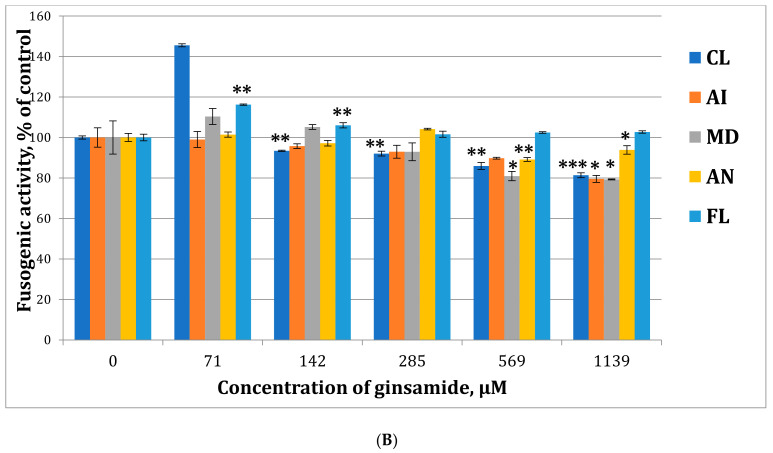
Hemaglutinin-inhibiting activity of ginsamide against the HA of influenza (**A**,**B**) viruses. Panel (**A**): Hemaglutinin-inhibiting activity of ginsamide against the HA of A/Puerto Rico/8/34 and A/Vladivostok/2/2009 (H1N1) viruses. Panel (**B**): Hemaglutinin-inhibiting activity of ginsamide against the HA of A/California/7/2009 (H1N1); A/Aichi/2/1968 (H3N2); A/Mallard/Pennsylvania/1984 (H5N2); A/Anhui/1/2013 (H7N9); B/Florida/4/2006 viruses. Ginsamide (0–1139 µM) was mixed with 128 hemagglutinating units of the corresponding influenza virus, incubated at room temperature for 30 min, and mixed with 0.75% chicken erythrocytes. Plates were incubated for 1 h at +4 °C, 150 µL of supernatant was removed, and 150 μL of MES buffer (0.1 M MES, 0.15 M NaCl, 0.9 mM CaCl_2_, 0.5 mM MgCl_2_, pH 5.0) was added, mixed and incubated for 1 h at 37 °C. After sedimentation of the erythrocytes at 1200 rpm for 6 min, the optical density in the wells was measured at 405 nm. The HA-inhibiting activity of the compounds was calculated as (ODc − ODb)/(ODp − ODb) × 100%, where ODp and ODcare the mean optical densities in the wells with PBS and the compound under investigation, respectively, and ODb (background) is the mean optical density in the wells with erythrocytes but without virus and compounds. The activity of HA in the control wells (no virus) was calculated by comparing it to the HA activity of the influenza A virus. PR: influenza A/Puerto Rico/8/34; VL: A/Vladivostok/2/2009 (H1N1); CL: A/California/7/2009 (H1N1); AI: A/Aichi/2/1968 (H3N2); MD: A/Mallard/Pennsylvania/1984 (H5N2); AN: A/Anhui/1/2013 (H7N9); FL: B/Florida/4/2006 viruses. * *p* < 0.05, ** *p* < 0.01, *** *p* < 0.001.

The inhibiting properties of the lead compound against fusogenic activity of viral HA have been evaluated in a fusion assay using chicken erythrocytes. As shown in Figure 6, only viruses of H1 subtype appeared susceptible to the compound. Of the three H1 viruses studied, the highest activity was demonstrated against A/Puerto Rico/8/34 and A/Vladivostok/2/09, while A/California/07/09 virus was less sensitive. Influenza viruses A of H3, H5, and H7 subtypes, as well as influenza virus B, did not decrease their fusogenic activity, or this effect was observed only at the highest concentrations of ginsamide and at a much lesser extent compared to H1N1 strains.

### 2.4. In Vivo Experiments

**Figure 7:**
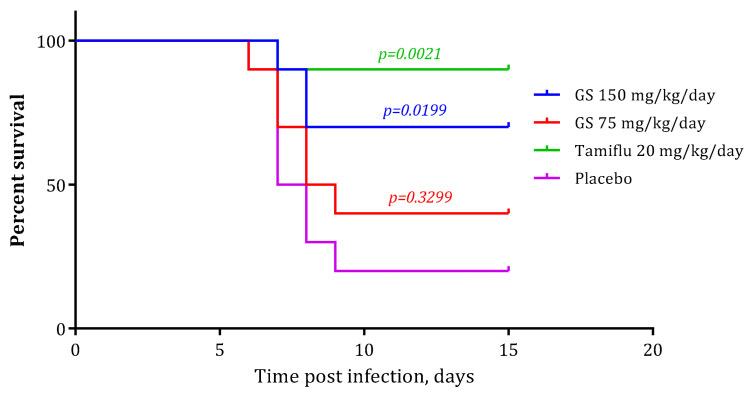
Dynamics of mortality of mice in the course of pneumonia caused by influenza virus A/Puerto Rico/8/34 (H1N1). Animals were infected intranasally with 2.5 × 10^4^ pfu of the virus per mouse and monitored for 15 days post-infection. GS was applied orally once a day at days −1, 0, 1, 2, 3, 4, and 5 post-infection. Log-rank test for comparison with A/PR/8/34 (H1N1)-infected group.

In order to comprehensively assess the protective activity of GS, we performed in vivo experiments using the model of lethal pneumonia in mice caused by influenza virus A/Puerto Rico/8/34 (H1N1). As the results suggest (Figure 7), GS significantly decreased mortality when administered at the highest dose (150 mg/kg/day, *p* = 0.0199). Decreasing the GS dose by 50%, from 150 to 75 mg/kg/day, resulted in non-significant protection of the animals (*p* = 0.3299). Thus, although highly effective in vitro, GS demonstrated relatively low activity on the animal model of influenza. We suggest that this may be due to the rather low bioavailability of the substance. In future work, we will select optimal drug forms that increase bioavailability and conduct more in-depth studies of activity using an animal model. In vivo toxicity studies were performed for compound **1a**, and it was shown that the LD_50_ of compound **1a** exceeds 5000 mg/kg weight, which confirms the low toxicity of these compounds.

### 2.5. Propagation with Ginsamide in Cell Culture Results in Selection of GS-Resistant Strains of Influenza A Virus

In order to assess the genetic barrier to resistance selection to GS, we passaged influenza virus A/Puerto Rico/8/34 (H1N1) in MDCK cells at increasing concentrations of GS and further evaluated the emerging resistance level, and identified the amino acid substitutions. After ten subsequent passages of egg-derived virus in cell culture, the IC_50_ to GS was determined as 23.6 μM, which was 155 times higher than the initial egg-propagated virus. After 10 passages in cells in the presence of GS, the virus demonstrated a level of susceptibility to GS with IC_50_ of 106.1 μM, i.e., almost five-fold higher than without GS and almost 700 times higher than the cell-propagated virus without GS. Ginsamide, therefore, stimulates the selection of resistance of influenza virus, suggesting direct antiviral activity and a virus-specific target.

**Figure 8:**
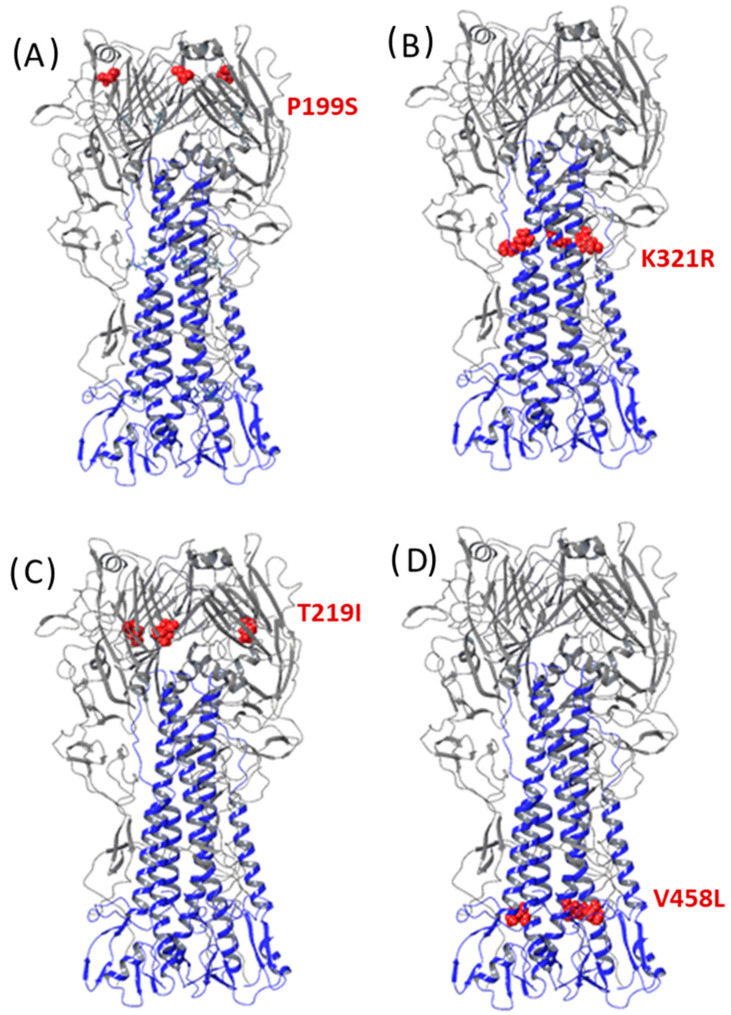
Localization of amino acid substitutions in GS-R virus compared to PR8 (GenBank: CY121109.1). (**A)** P199S; (**B**) K321R; (**C**) T219I; (**D**) V458L.

After a resistant variant of the virus was obtained, viruses were plaque purified and HA segments of three clones from each virus—initial (embryonal), control (C, propagated in cells without GS), and GS-resistant (GS-R, propagated with GS)—were sequenced to localize amino acid substitutions. After a comparison of the sequences, several substitutions were identified (Figure 8). Firstly, the P199S substitution was found in all clones of both C and GS-R viruses, suggesting that this substitution demonstrates the adaptation of the virus grown in chicken embryos to mammalian cells. This was confirmed by further localization of this position in the receptor-binding domain of the HA (Figure 8A). Secondly, three substitutions were identified in the GS-R virus compared to the C virus: K321R, T219I, and V458L (Figure 8B–D). Among these three, one (T219I) was located in the HA1 subunit at the receptor-binding site, one (K321R) was located near the fusion peptide, between the HA1 and HA2 subunits, and one (V458L) was located in the stem of HA, close to the proteolytic site.

**Figure 9:**
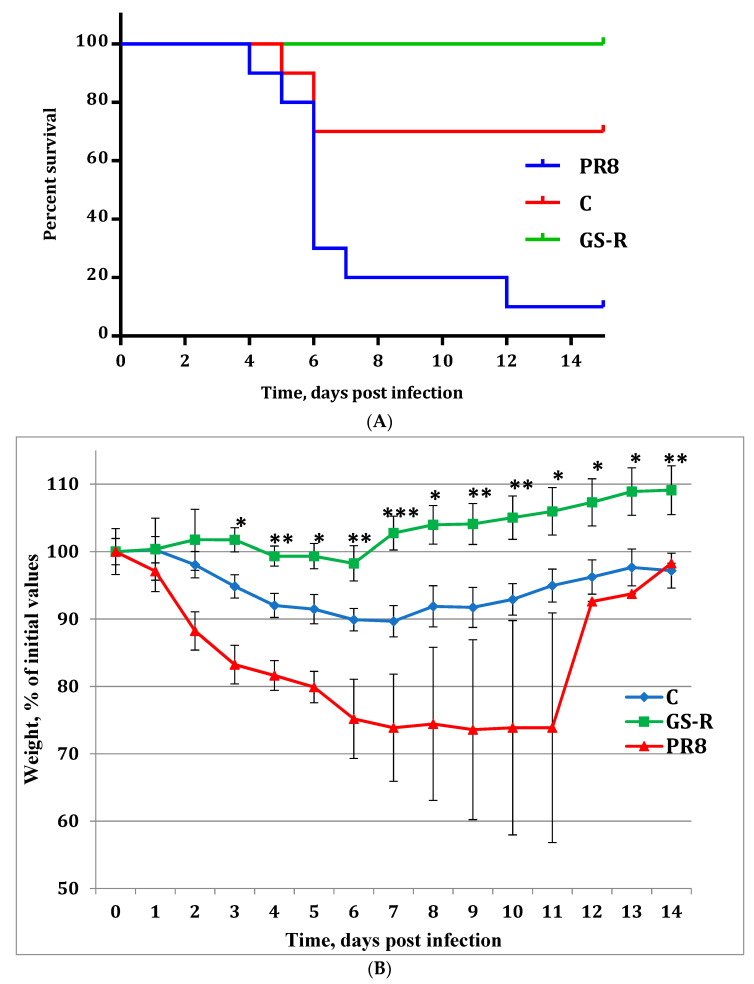
Effect of the development of GS-resistance on the pathogenicity of influenza virus A/Puerto Rico/8/34 (H1N1). Animals (ten per group) were intranasally infected with 5 × 10^4^ TCID_50_ per mouse of initial, egg-derived virus (PR8), virus after ten passages in MDCK cells (C), or virus after ten passages in MDCK cells in the presence of GS (GS-R). Animals were monitored daily for lethality and weight. (**A**) dynamics of mortality in the course of influenza pneumonia; (**B**) weight dynamics. Asterisks indicate the level of significance after comparison between C and GS-R viruses. * *p* < 0.05, ** *p* < 0.01, *** *p* < 0.001.

For further characterization of the biological properties of the GS-R virus, we assessed its pathogenicity in mice in comparison with PR8 and C viruses. For this purpose, animals were infected with equal doses of one of these three viruses and monitored daily for mortality and body weight. The results are summarized in Figure 9.

As can be seen from the data presented (Figure 9A), the adaptation of the egg-derived virus to mammalian cells in the course of passaging in the culture resulted in a drop in pathogenicity. Indeed, in the group infected with the PR8 virus, nine of the ten infected animals died, while the mortality among mice infected with the same amount of control virus propagated in MDCK cells was only 3 out of 10 mice (*p* = 0.0116, Mantel-Cox test).

In the GS-R group (animals inoculated with virus cultivated with GS), no mortality was observed at all (*p* < 0.0001, Mantel-Cox test). At the same time, mortality in the C and GS-R groups was statistically indistinguishable (*p* = 0.0675, Mantel-Cox test). Thus, the influenza virus with developed GS resistance demonstrates a tendency to lower the pathogenicity to animals compared to the control virus propagated without GS.

These data were confirmed by analysis of weight dynamics in the experimental groups (Figure 9B). In accordance with previous results, both viruses propagated in MDCK cells appeared less pathogenic than the initial, egg-derived virus. Nevertheless, animals infected with the GS-R virus demonstrated less weight loss than mice infected with the C virus. Thus, the cultivation of influenza virus in the presence of GS leads to attenuation of the virus and loss of its pathogenicity.

**Figure 10:**
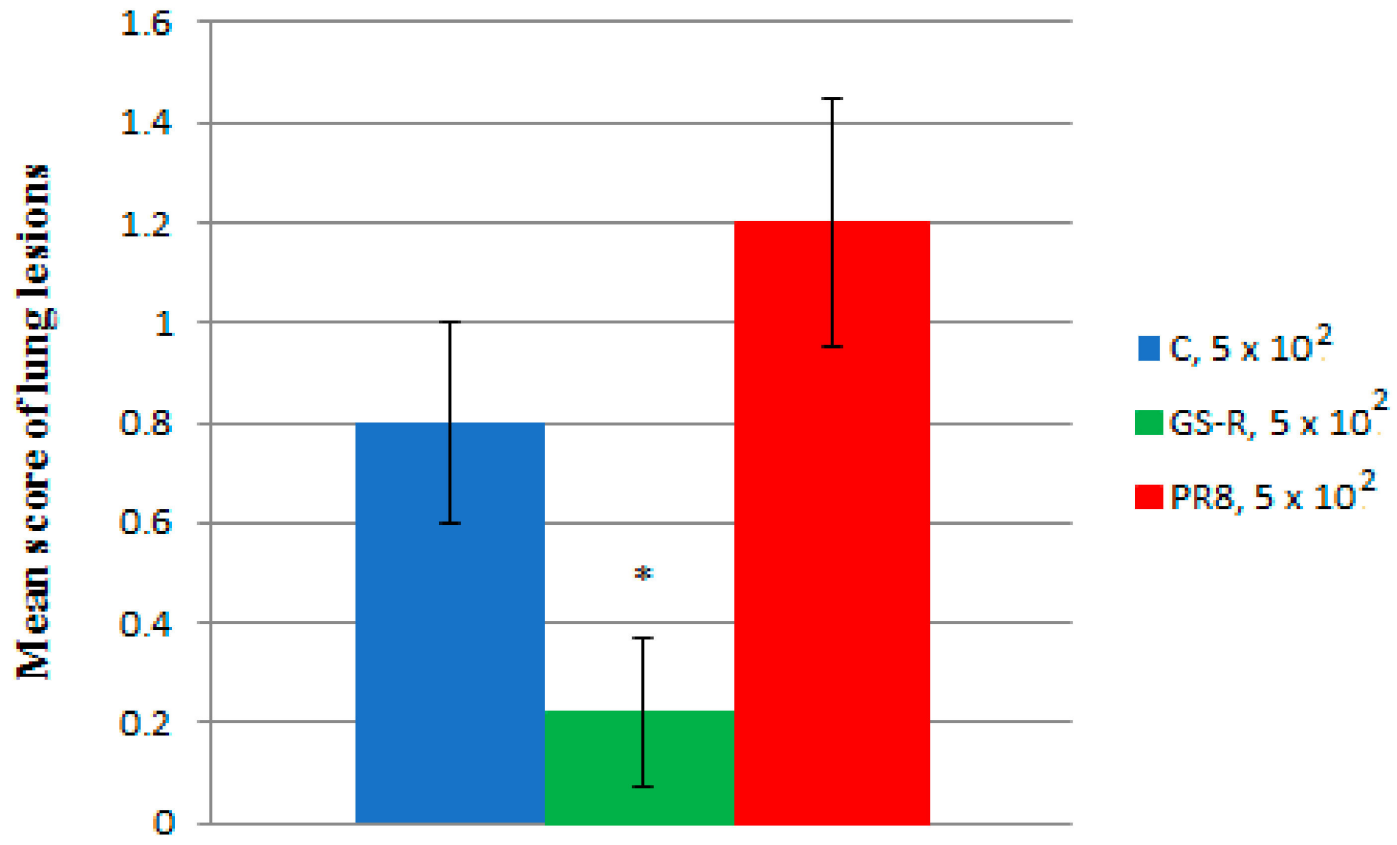
Development of post-influenza lesions in lungs of mice infected with three influenza viruses. Animals were inoculated with influenza viruses (5 × 10^2^ TCID_50_ per mouse), and their lungs were studied after 10 days for signs of post-influenza lesions. PR8: initial egg-derived virus, C: virus after ten passages in MDCK cells; GS-R: virus after ten passages in MDCK cells in the presence of ginsamide, * represents *p* < 0.05.

In order to additionally evaluate the pathogenic properties of these three viruses, three separate groups of animals were inoculated with low infecting doses (5 × 10^2^ TCID_50_ per mouse), and their lungs were studied after 10 days for signs of post-influenza lesions (Figure 10). No mortality was observed at this infecting dose. As can be seen from the results presented, the mean score of lung lesions in the PR8 group was 1.2. Infection with both C and GS-R viruses resulted in more moderate lesions. In accordance with the previous results, the GS-R virus demonstrated the lowest pathogenicity (mean score 0.2, *p* = 0.0044 vs. PR8, *p* = 0.033 vs. C). The control virus that was propagated in MDCK cells without GS demonstrated lower pathogenicity than PR8 (mean score 0.8), although it was statistically identical to the PR8 group (*p* = 0.2277).

The results presented suggest that the GS-resistance of influenza virus emerged and developed in cell culture relatively easily. Indeed, influenza virus drug resistance has been developed against the camphor derivative camfecene after six passages [43]. The development of drug resistance to the M2 inhibitor amantadine has been shown after 2–4 passages [44], to the neuraminidase inhibitor oseltamivir after five passages [45], and to the neuraminidase inhibitor zanamivir after 14 passages [46]. In the case of GS, this took 10 sequential passages in cell culture, with a gradual increase of IC_50_ values. The resulting strain demonstrated a high level of resistance to GS, with an IC_50_ value 700 times higher than the initial virus.

Surprisingly, passaging of influenza virus in MDCK cells resulted in a decrease of virus susceptibility to GS even in the control virus, when no GS was added into the culture medium. Although this was even more pronounced in the presence of GS, this phenomenon can be explained by several modes of action of GS, including several binding sites that can differently participate in viral replication in eggs and MDCK cells. Indeed, the conditions of virus cultivation in these two systems differ in terms of receptor binding, which are different in avian and mammalian cells. In addition, the proteases involved in HA activation are different in eggs and cell culture, being represented only by trypsin in the latter case. This can be indirectly confirmed by resistance-associated amino acid substitutions scattered at different functional domains of HA (Figure 8).

Fusion proteins of enveloped viruses represent attractive targets for the development of novel antivirals. These proteins are essential for a normal virus life cycle, and they act in their early phases. Their inhibitors, therefore, can potentially prevent the infection of cells, giving the host an opportunity to maintain a full immune response. However, structural differences among fusion proteins of viruses of different families do not allow the development of broad-range inhibitors. Even in the case of the influenza A virus, according to the structural specificity of the fusion peptide site, the HAs of the influenza virus are divided into two groups: I and II [47]. In the vast majority of cases, fusion inhibitors suppress viruses bearing HAs of either group I or group II [48,49,50,51,52]. In the case of GS, this specificity appeared to be even more pronounced, with only viruses of the H1 subtype being susceptible to the compound. These features may reflect its specific sites of HA binding, which are structurally different in different HA subtypes.

The results of in vivo experiments demonstrate that GS is effective only when applied at high doses. This can be explained by the low bioavailability or fast decomposition of the compound. Further attempts are, therefore, needed to increase its half-life time and optimize its pharmacokinetic parameters. This could be achieved by either optimization of the application schedule or searching for other chemical derivatives, or pro-drugs, with a prolonged period of activity.

After cultivation in MDCK cells, either in the presence or absence of GS, a loss of viral pathogenicity was observed. This effect was previously described for highly pathogenic avian influenza virus [53], and our data, therefore, corresponds to the previously obtained results. However, this effect was much more pronounced for the GS-resistant virus, whose virulence was much lower compared to the control virus grown without GS. This was manifested in lower mortality, lower weight loss, and a considerable reduction in the extent of post-influenza lung lesions. Previously, we described a similar phenomenon for yet another effective anti-influenza cage compound, camphecene [43], which, similar to ginsamide, inhibits the fusogenic activity of viral HA. Taken together, these results may suggest that this is a common feature for compounds of similar structure, and further attempts should be undertaken to completely decipher the mechanisms of the relationship between the resistance to HA blockers and the virulence of resistant strains.

### 2.6. Molecular Modeling Study

**Figure 11:**
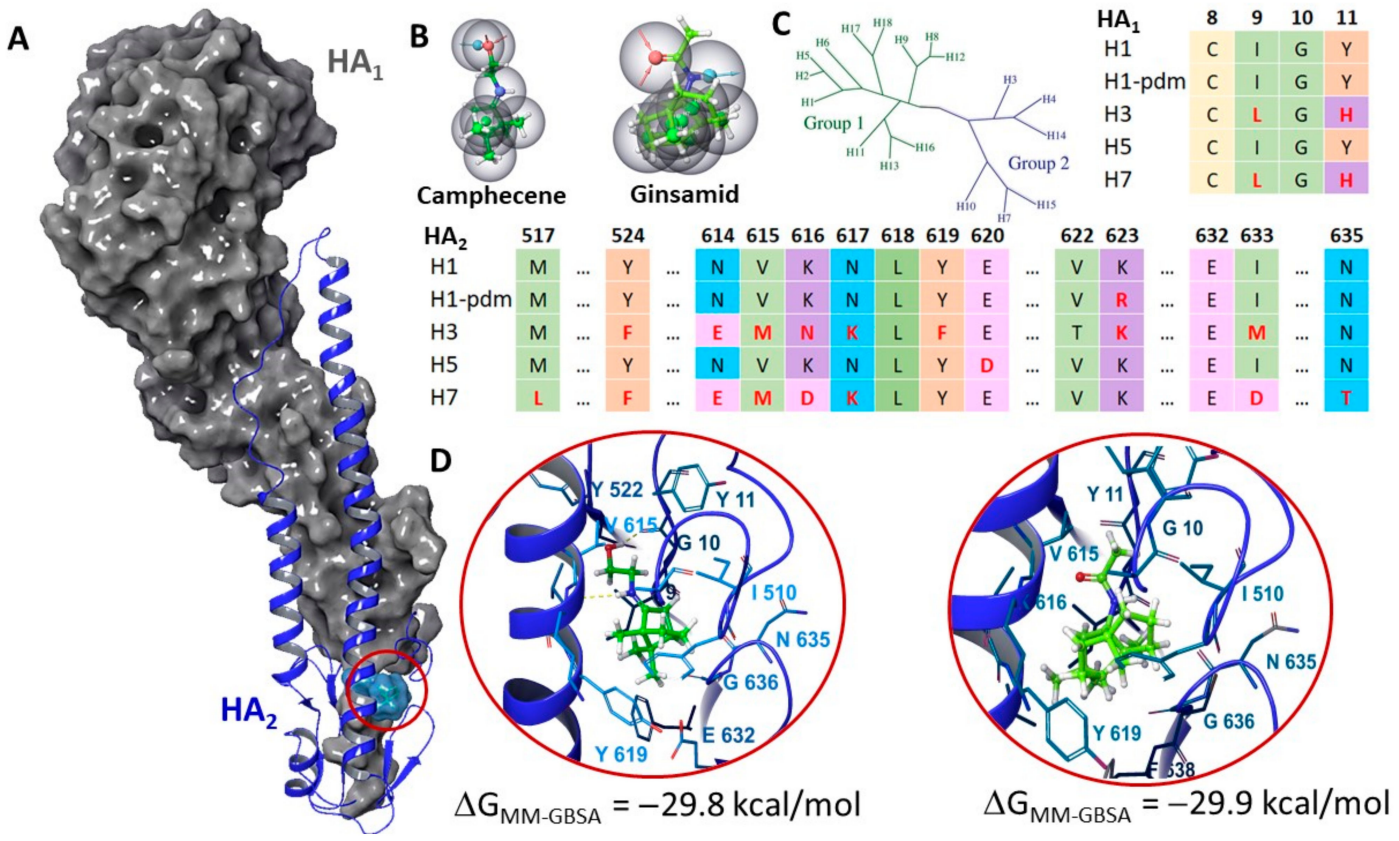
Molecular modeling results: (**A**): Monomer of HA: gray surface corresponds to HA1; blue secondary structure: HA2. (**B**): Pharmacophore profiles of Camphecene and Ginsamide: hydrophobic descriptors are represented by green balls; acceptor—by red, and donor—by light-blue ones; (**C**): Phylogenetic tree of influenza A HAs [47] and results of binding site alignment procedure H1 (PBD code 1RU7 [55]; H1-pdm (PBD code 3LZG [58]); H3 (pdb code 3EYM [47]); H5 (PDB code 6CF5 [59]) and H7 (PBD code 6ID2 [60]). Amino acids differences are written in red letters. (**D**): Molecular docking result: H-bonds are represented with yellow dotted lines.

According to [43,54] Camphecene and its analogs can bind in the small hydrophobic pocket located in the place of proteolysis between the long α-helix and two-loop N-terminate of HA2 (Figure 11A). The potential site of ginsamide binding was chosen for two reasons: first, the similarity of the pharmacophore profiles of camphecene and ginsamide (Figure 11B), and second, the result of genome-wide sequencing of the GS-resistant influenza A virus. Serial passaging of influenza in the presence of ginsamide leads to the causes of a mutation in HA V458L (amino acids number V615L correspond to 1RU7 [55] PDB code [56]. It has been previously shown that Camphecene may cause the same mutation. Moreover, Ginsamide exhibits pronounced antiviral activity against influenza A/H1N1 strains (Table 2) as well as Camphecene [57]. The activity of Ginsamide against H3N2, H5N2, and H7N9 virus strains is less pronounced. This can be explained by the difference in the amino acid sequence of the potential HA binding site of different phylogenetic groups. In particular, in HA2 of the first group, which include H1, H5, valine is located at position 615 (Figure 11C), and methionine is located at the second (H3 and H7).

Ginsamide binds at the binding site (Figure 11D) like camphecene, forming a series of strong hydrophobic contacts with surrounding amino acids. Unlike camphecene, ginsamide is probably not protonated. The binding energies of the ligand and the protein in the ligand-protein complex are comparable for both ligands.

## 3. Materials and Methods

### 3.1. Chemistry

#### 3.1.1. General Information

Reagents and solvents were purchased from commercial suppliers and used as received. Dry solvents were obtained according to standard procedures. Reactions monitoring, the content of the compounds in fractions during chromatography, and the purity of the target compounds were determined using 7890A gas chromatograph (Agilent Tech., Santa Clara, CA, USA) with an Agilent 5975C quadrupole mass spectrometer as detector; HP-5 capillary column, He as carrier gas (flow rate 2 mL/min, flow division 99:1). ^1^H and ^13^C-NMR spectra were recorded on Bruker spectrometers (Bruker BioSpin GmbH, Ettlingen, Germany), including an AV-300 instrument at 300.13 MHz (^1^H) and 75.47 MHz (^13^C), an AV-400 instrument at 400.13 MHz (^1^H) and 100.61 MHz (^13^C), and a DRX-500 instrument at 500.13 MHz (^1^H) and 125.76 MHz (^13^C) in CDCl_3_; chemical shifts δ were report ed in ppm relative to residual CHCl_3_ (d(CHCl_3_) 7.24, d(CDCl_3_) 76.90 ppm), *J* in Hz. High-resolution mass spectra (HRMS) were obtained with a DFS Thermo Scientific mass spectrometer in a full scan mode (0–500 *m*/*z*, 70 eV electron impact ionization, direct sample administration). We used caryophyllene [α${]}_{580}^{20}$−13.8 (CHCl_3_, c 4.3), isocaryophyllene [α${]}_{580}^{20}$−20.0 (CHCl_3_, c 5.4) obtained by isomerisation of caryophyllene according to [42]. Synthesis, spectral characteristics, and X-ray studies of agent **1a** have been previously described [37].

#### 3.1.2. General Procedure for Synthesis of Derivatives **1b**–**d**

To a solution of 0.5 g caryophyllene in 5 mL of appropriate nitrile was added 0.2 mL sulfuric acid, stirred for 40 to 60 min, the reaction mixture was neutralized with saturated Na_2_CO_3_ solution, extracted with methylene chloride, the organic extract was washed with water, dried (MgSO_4_). The mixture of reaction products was separated by column chromatography on SiO_2_ (100–160, PMFD) (eluent-hexane with a gradient of diethyl ether from 0.5% to 10%). The isomerisation products were separated using a non-polar eluent. The chromatography was controlled by GC/MS analysis of the fractions.

*N-(2,2,4,7a-tetramethyloctahydro-3aH-1,4-ethanoinden-3a-yl)propionamide* (**1b**). The compound was obtained from the reaction of isocaryophyllene with propiononitrile as a white powder, yield after chromatography 61%, (purity 97%). M.p. 103 °C. IR (KBr, cm^−1^): ν 3330 (NH), 1650 (C=O). UV (MeOH), λmax/nm: 202. ^1^H-NMR (CDCl_3_) δ (ppm): 0.81 (3H, s, Me-12), 1.05 (3H, s) and 1.20 (3H, s, Me-13 and Me-14), 1.11 (3H, s, Me-15), 1.12 (3H, t, *J* = 7.6, H-18), 2.17 (2H, q, *J* = 7.6, H-17), 2.30 (1H, d, *J* = 15.1) and 2.40 (1H, d, *J* = 15.1, H-6), 5.28 (1H, s, NH), 1.81-1.96 (3H, m, H-10, H-9, H-3), 1.44–1.66 (4H, m, H-10, H-3, H-11, H-2), 1.37–1.41 (1H, m, H-4), 1.22–1.33 (2H, m, H-2, H-9), 1.09–1.16 (1H, m, H-11). ^13^C-NMR (CDCl_3_) δ (ppm): 172.9 (C-16), 56.1 (C-4), 26.9 (C-12), 30.7 (C-15), 28.2 and 34.0 (C-13 and C-14), 10.2 (C-18), 21.5 (C-10), 25.9 (C-3), 33.6 (C-2), 30.7 (C-17), 67.6 (C-7), 33.9 (C-9), 34.9 (C-11), 36.5 (C-5), 40.2 (C-1), 45.1 (C-6), 45.9 (C-8). HRMS (ESI): *m*/*z* calcd for C_18_H_31_NO: 277.2400; found 277.2402.

*N-(2,2,4,7a-tetramethyloctahydro-3aH-1,4-ethanoinden-3a-yl)isobutyramide* (**1c**). The compound was obtained from the reaction of isocaryophyllene with isobutyronitrile as a white powder, yield after chromatography 68%, (purity 99%). M.p. 103 °C. IR (KBr, cm^−1^): ν 3320 (NH), 1645 (C=O). UV (MeOH), λmax/nm: 202. ^1^H-NMR (CDCl_3_) δ (ppm): 0.82 (3H, s, Me-12), 1.06 (3H, s) and 1.21 (3H, s, Me-13 and Me-14), 1.13 (3H, s, Me-15), 1.13 (3H, d, *J* = 7.0) and 1.14 (3H, d, *J* = 7.0, Me-18 and Me-19), 2.30 (1H, sept, *J* = 7.0, H-17), 2.30 (1H, d, *J* = 15.1) and 2.40 (1H, d, *J* = 15.1, H-6), 5.30 (1H, s, NH), 1.82–1.98 (3H, m, H-10, H-9, H-3), 1.44–1.68 (4H, m, H-10, H-3, H-11, H-2), 1.37–1.43 (1H, m, H-4), 1.24–1.35 (2H, m, H-2, H-9), 1.08–1.19 (1H, m, H-11). ^13^C-NMR (CDCl_3_) δ (ppm): 176.1 (C-16), 56.1 (C-4), 19.8 and 20.0 (C-18 and C-19), 36.7 (C-17), 26.8 (C-12), 30.7 (C-15), 28.2 and 33.9 (C-13 and C-14), 21.5 (C-10), 26.0 (C-3), 33.7 (C-2), 67.3 (C-7), 34.0 (C-9), 35.0 (C-11), 36.5 (C-5), 40.2 (C-1), 45.1 (C-6), 46.0 (C-8). HRMS (ESI): *m*/*z* calcd for for C_19_H_33_NO: 291.2557; found 291.2561.

*N-(2,2,4,7a-tetramethyloctahydro-3aH-1,4-ethanoinden-3a-yl)benzamide* (**1d**). The compound was obtained from the reaction of isocaryophyllene with benzonitrile as a light yellow oil, yield after chromatography 32%, (purity 96%). IR (KBr, cm^−1^): ν 3338 (NH), 1660 (C=O). UV (MeOH), λmax/nm: 200, 225. ^1^H-NMR (CDCl_3_) δ (ppm): 7.65–7.74 (2H, m, H-18, H-22), 7.35–7.49 (3H, m, H-19, H-20, H-21), 6.05 (1H, s, NH), 0.92 (3H, s, Me-12), 1.11 (3H, s) and 1.25 (3H, s, Me-13 and Me-14), 1.23 (3H, s, Me-15), 2.54 (1H, d, *J* = 15.0) and 2.46 (1H, d, *J* = 15.0, H-6), 1.79–2.04 (3H, m, H-10, H-9, H-3), 1.49–1.76 (4H, m, H-10, H-3, H-11, H-2), 1.44–1.49 (1H, m, H-4), 1.31–1.43 (2H, m, H-2, H-9), 1.01–1.08 (1H, m, H-11). ^13^C-NMR (CDCl_3_) δ (ppm): 166.8 (C-16), 136.4 (C-17), 130.8 (C-20), 128.4 (C-19, C-21), 126.4 (C-18, C-22), 56.1 (C-4), 27.0 (C-12), 31.0 (C-15), 28.2 and 34.0 (C-13 and C-14), 21.5 (C-10), 68.2 (C-7), 25.9 (C-3), 33.7 (C-2), 34.2 (C-9), 35.1 (C-11), 36.5 (C-5), 40.5 (C-1), 45.0 (C-6), 46.2 (C-8). HRMS (ESI): *m*/*z* calcd for C_22_H_31_NO: 325.2400; found 325.2405.

#### 3.1.3. Synthesis of an Amine 2 from Ginsamide

The sodium hydride was washed with hexane (4 × 20 mL), dried on a rotary evaporator. Dimethylformamide (20 mL) was added to the dried sodium hydride (1.0 g, 0.0382 mol), dried over 4 Å molecular sieves. Then malone ether (1.33 g, 0.0831 mol) was carefully added to the mixture in small portions. After stopping the rapid hydrogen evolution, solid Ginsamide (1.0 g, 0.0038 mol) was added. The reaction mixture was stirred in an argon atmosphere at 135–145 °C for 8 h. After cooling down, 25 mL ethyl acetate was added, washed with 5% sodium chloride solution (4 × 10 mL), the organic part was dried over MgSO_4_, the drying agent was filtered off, the solvent was removed, 0.85 g of dark oil was obtained. To a solution of the crude product in 10 mL acetonitrile, an ether solution of hydrochloric acid was added until a strongly acidic reaction with an indicator paper. After distillation of the solvent, the solid product was recrystallized from acetonitrile/ethyl alcohol (1:1). After drying in an oil pump vacuum, a white crystalline solid of 0.626 g (64% yield, purity 99%) was obtained.

*2,2,4,7a-tetramethyloctahydro-3aH-1,4-ethanoinden-3a-amine hydrochloride* (**2**). Mp 257.8–261.3 °C. Found (%): C 70.01, H 10.86, Cl 13.71, N 5.41. C15H28ClN. Calculated (%): C 69.87, H 10.95, Cl 13.75, N 5.43. UV (MeOH), λmax/nm: 202. ^1^H-NMR (DMSO-*d*_6_) 1.99 (1H, d, *J =* 14.3) and 1.88 (1H, d, *J =* 14.3, H-6), 0.87 (3H, s, Me-12), 1.00 (3H, s) and 1.22 (3H, s, Me-13 and Me-14), 1.22 (3H, s, Me-15), 7.85 (3H, s, NH2.HCl), 1.14-1.25 (1H, m, H-11), 1.84-1.94 (1H, m, H-3), 1.31-1.52 (5H, m, 2H-9, H-4, H-10, H-3), 1.58-1.82 (4H, m, H-11, H-10, 2H-2). ^13^C-NMR: (DMSO-*d*_6_) 67.7 (C-7), 56.7 (C-4), 44.5 (C-6), 45.0 (C-8), 36.3 (C-5), 37.0 (C-1), 25.2 (C-12), 29.4 (C-15), 28.2 and 34.0 (C-13 and C-14), 21.1 (C-10), 25.7 (C-3), 32.0 (C-2), 33.5 (C-11), and 33.8 (C-9).

#### 3.1.4. Synthesis of a Thioamide **3a**

To a solution of **1a** (2.10 mmol) in dry toluene (10 mL) was added Lawesson’s reagent (1.155 mmol) in one portion. After stirring for 2 h, the reaction mixture was quenched with water and extracted with diethyl ether (2 × 15 mL). The combined organic layers were dried over MgSO_4_ and evaporated to dryness. The residue was purified by column chromatography on silica gel eluting with Et_2_O/petroleum ether (1:2) to give the pure **3a** (58% yield) as a light yellow solid.

*N-(2,2,4,7a-tetramethyloctahydro-3aH-1,4-ethanoinden-3a-yl)ethanethioamide* (**3a**) IR (KBr, cm^−1^): ν 3317 (NH). UV (MeOH), λmax/nm: 195. ^1^H-NMR (DMSO-*d*_6_) δ (ppm): 0.91 (3H, s, Me-12), 1.04 (3H, s) and 1.20 (3H, s, Me-13 and Me-14), 1.10 (3H, s, Me-15), 2.50 (3H, s, Me-17), 2.73 (1H, d, *J =* 15.1) and 2.44 (1H, d, *J =* 15.1, H-6), 8.62 (1H, s, NH), 1.75–1.98 (3H, m, H-10, H-9, H-3), 1.33–1.38 (1H, m, H-4), 1.42–1.59 (4H, m, H-10, H-3, H-11, H-2), 1.22–1.33 (2H, m, H-2, H-9), 1.06–1.11 (1H, m, H-11). ^13^C-NMR (DMSO-*d*_6_) δ (ppm): 200.3 (C-16), 55.7 (C-4), 36.4 (C-17), 28.4 (C-12), 29.6 (C-15), 28.8 and 34.2 (C-13 and C-14), 21.2 (C-10), 25.4 (C-3), 31.3 (C-2), 33.6 (C-9), 33.8 (C-11), 35.9 (C-5), 72.7 (C-7), 41.8 (C-1), 45.2 (C-6), 46.3 (C-8). HRMS (ESI): *m*/*z* [M − H]^+^ calcd for C_17_H_28_NS: 278.1937; found 278.1941.

### 3.2. Biological Studies

#### 3.2.1. Viruses and Cells

Influenza viruses A/Puerto Rico/8/34 (H1N1), A/California/07/09 (H1N1)pdm09, A/Aichi/2/68 (H3N2), A/mallard/Pennsylvania/10218/84 (H5N2), A/Vladivostok/2/2009 (H1N1) (oseltamivir-resistant), A/Anhui/1/2013 (H7N9), and B/Florida/4/2006 (Yamagata-like) were obtained from the collection of viruses of St. Petersburg Pasteur Institute. Prior to the experiment, viruses were propagated in the allantoic cavity of 10–12 day old chicken embryos for 48 h at 36 °C (influenza A viruses) or 72 h at 36 °C (influenza B virus). Infectious titer of the virus was determined in MDCK cells (ATCC # CCL-34) in 96-wells plates in alpha-MEM medium with 10% fetal bovine serum.

#### 3.2.2. Animals

Inbred female BALB/c mice, 6–8 weeks old, were obtained from the animal breeding facility of the Russian Academy of Medicine “Rappolovo” (Rappolovo, Russia). The mice were quarantined 48 h prior to the experimental manipulation and were fed standard rodent chow, and had *ad libitum* access to water. Animal experiments were conducted in accordance with the principles of laboratory animal care (Guide for the Care and Use of Laboratory Animals, National Academy Press, Washington, DC, USA, 1996) and approved by the Institutional Ethical Committee (approval number 46).

#### 3.2.3. Cytotoxicity Assay

MDCK cells were seeded onto 96-well culture plates (10^4^ cells per well) and incubated at 36C in 5% CO_2_ until continuous monolayer formation. To assess the toxicity of compounds, a series of their three-fold dilutions at concentrations of 300 to 4 μg/mL in Eagle’s MEM medium were prepared. The dilutions were added to the wells of the plates. Cells were incubated for 72 h at 36 °C in a CO_2_ incubator under 5% CO_2_. Further, a microtetrazolium (MTT) assay was performed on 96-well plates. The cells were washed 2 times with saline (0.9% NaCl) and 100 μL/well of MTT solution (3-(4,5-dimethylthiazol-2-yl)-2,5-diphenyltetrazolium bromide) at a concentration of 0.5 μg/mL in MEM was added. The plates were incubated for 1 h at 36 °C, the liquid was removed and DMSO (0.1 mL per well) was added. The optical density of the cells was measured on a Thermo Multiskan FC spectrophotometer (Thermo Fisher Scientific, Waltham, MA, USA) at a wavelength of 540 nm. Based on the obtained data, the CC_50_, the concentration of the compound that destroys 50% of the cells in culture, was calculated for each specimen.

#### 3.2.4. CPE Reduction Assay

The compounds in appropriate concentrations were added to MDCK cells (0.1 mL per well). MDCK cells were further infected with either A/Puerto Rico/8/34 (H1N1) or A/Anhui/1/2013 (H7N9) influenza virus (MOI 0.01). Plates were incubated for 72 h at 36 °C at 5% CO_2_. After that, cell viability was assessed by MTT test as described above. The cytoprotective activity of compounds was considered as their ability to increase the values of OD compared to control wells (with virus only, no drugs). Based on the results obtained, the values of IC_50_, i.e., the concentration of compounds that results in 50% cells protection, were calculated using GraphPad Prism software. Values of IC_50_ obtained in micrograms/mL were then calculated into micromoles. Based on the obtained data, the selectivity index (SI), the ratio of CC50 to IC50, was calculated for each compound.

#### 3.2.5. Virus Titration and Virus Yield Reduction Assay

The lead compound Ginsamide in appropriate concentrations was dissolved in MEM with 1 µg/mL trypsin and incubated with MDCK cells for 1 h at 36 °C. Each concentration of the compound was tested in triplicate. The cell culture was then washed twice with phosphate-buffered saline (PBS) and incubated with influenza virus A/Puerto Rico/8/34 (H1N1) (MOI 0.01) for 1 h. The monolayers were washed twice with PBS, and the same compound-containing medium was added. The plates were incubated for 24 h at 36 °C in the presence of 5% CO_2_. A virus titer in the supernatant was further determined by TCID_50_ assay by MTT test [61] after cultivating the virus in MDCK cells for 72 h at 36 °C in the presence of 5% CO_2_. For calculations, virus titer was expressed as a percentage of the titer in control wells without compounds. The 50% inhibiting concentration (IC_50_) of the drug, that are, the concentrations at which the virus production decreased two-fold, and the selectivity index (the ratio of CC_50_ to IC_50_) were calculated from the data obtained.

#### 3.2.6. Time-of-Addition Experiments

To determine the stage of the viral life cycle that is affected with the compound, cells were seeded into 24-wells plates and incubated with influenza virus A/Puerto Rico/8/34 (H1N1) (MOI 10) for 1 h at 4 °C. After washing of non-absorbed virions for 5 min with MEM, plates were incubated for 8 h at 36 °C at 5% CO_2_. The starting point of this incubation was referred to as 0 h. Ginsamide (final concentration 200 micromol/L) was dissolved in MEM, and cells were treated with ginsamide for the time periods as follows: (−2)–(−1) (before infecting); (−1)–0 (simultaneously to absorbtion); 0–2; 2–4; 4–6; 6–8 h post-infection (hpi). The treatment (−2)–8 hpi was considered as a positive control. In each case, after incubation, the compound was removed, and cells were washed for 5 min with MEM. After 8 h of growth, the infectious titer of the virus was determined in the culture medium and cells as described above.

#### 3.2.7. Haemolysis Assay

The membrane-disrupting activity of viral hemagglutinin was measured according to Maeda and Ohnishi [62] with slight modifications. Briefly, chicken erythrocytes were washed twice with PBS and resuspended to make a 0.75% (*vol/vol*) suspension in PBS, which was stored at 4 °C until use. One hundred microliters of ginsamide diluted in PBS to appropriate concentrations was mixed with an equal volume of the influenza virus (64 hemagglutinating units per 0.1 mL), or PBS for negative control. After incubating the virus-compound mixture at room temperature for 30 min, 50 µL of the mixture was transferred into U-bottom well and mixed with 150 µL of 0.75% chicken erythrocytes. Plates were incubated for 1 h at +4 °C for sedimentation and absorption of virions on erythrocytes. 150 µL of supernatant was then removed, 150 µL of MES buffer (0.1 M MES, 0.15 M NaCl, 0.9 mM CaCl_2_, 0.5 mM MgCl_2_, pH 5.0) was added, mixed, and incubated for 1 h at 37 °C for HA acidification and hemolysis. To separate nonlysed erythrocytes, plates were centrifuged at the end of incubation at 1200 rpm for 6 min. After sedimentation of erythrocytes 100 µL of supernatant were transferred into the wells of the fresh plate, and optical density in the wells was measured at 405 nm. The activity of compounds was considered as their ability to suppress the destruction of membranes and thus decrease the concentration of free hemoglobin and optical density in the wells compared to the control wells without additives. The activity of HA was calculated as (ODc − ODb)/(ODp − ODb) × 100%, where ODp and ODs are mean optical densities in the wells with PBS and compound under investigation, correspondingly, and ODb (background) is mean optical density in the wells with erythrocytes but without virus and compounds. The activity of HA in control wells without any virus was calculated compared to HA activity of influenza A virus.

#### 3.2.8. Hemagglutination Inhibition Assay

In order to assess the ability of compounds to interfere directly with HA receptor binding, we performed hemagglutination inhibition test. Two-fold dilutions of influenza A/Puerto Rico/8/34 virus-containing culture medium (1:8 to 1:256) were mixed with compounds at subtoxic concentrations and incubated for 1 h at 36 °C at 5% CO_2_ followed by adding an equal volume of 1% chicken erythrocytes. After incubation for 1 h at 20 °C the results were checked visually. Anti-hemagglutinin activity was evaluated by the ability of specimens to prevent virus-driven hemagglutination.

#### 3.2.9. In Vitro Selection and Analysis of Resistant Mutants

In order to study the development of resistance to Ginsamide, influenza virus A/Puerto Rico/8/34 (H1N1) (PR8) was serially passaged in MDCK cells in the presence of increased concentrations of the compound. Cells were infected with the virus and incubated for 3–5 days at 36 °C with 5% CO_2_ until a cytopathic effect was observed. The culture supernatants were centrifuged, and aliquots were used for sequential selection. In total, ten passages have been performed. One passage was performed with GS concentration of 0.5 μg/mL following by four passages at 1 μg/mL and further five passages with two-times increase of GS concentration from 2 to 32 μg/mL. The control virus (C) was passaged in MDCK cells in the absence of the agent. The values of IC_50_ for control and GS-treated virus (GS-R) were further studied by virus yield reduction assay. After six passages, three viruses (embryonal, C, and GS-R) were plaque-purified, and HA genes of three clones from each virus were sequenced. Viral RNA was extracted using the kit RNAzol^®^ (“GibcoBRL”). After reverse transcription and amplification of cDNA using HA-specific primers, PCR products were analyzed by automatic system MegaBACE 1000 DNA Analysis System using BrilliantDye™ Terminator, v 3.1 kit, and NimaPOP 7 polymer. The following sequencing primers were used: Seq_F1 GCAGGGGAAAATAAAAACAACC, Seq_R1 TTCAGCTTTGGGTATGAGCC, Seq_F2 AGCTCATGGCCCAACCAC, Seq_R2 GGATTGGATGGACGGAATGTT, Seq_F3 CACCAATGTATGCTTTCGCAC, Seq_R3 GCATTATATGTCCAAATGTCCA, Seq_F4 TCCAGAGGTCTATTTGGAGCC, Seq_R4 TTCTGAAATTCTAATCTCAGATGCA. The chromatograms were converted into contigs using DNA Baser Sequence Assembler program. The sequences were aligned to a reference PR8 HA sequence (GenBank: CY121109.1). The nucleotide sequences were translated into amino acids by free online software (https://web.expasy.org/translate/) (accessed on 12 September 2021).

#### 3.2.10. In Vivo Experiments

In order to evaluate anti-influenza activity of ginsamide in vivo, mice (10 per group) were infected with 5 × 10^4^ TCID_50_ of the virus per mouse. Ginsamide (final dose 75 or 150 mg/kg body weight per day) was applied orally via gavage once a day in a volume of 0.2 mL for days 1 to 5 post-infection (p.i.) starting 1 h post-infection. The reference drug oseltamivir phosphate (Tamiflu, final dose 20 mg/kg body weight per day) was dissolved in saline and applied to mice orally via gavage once a day in a volume of 0.2 mL for days 1 to 5 post-infection (p.i.) starting 1 h post-infection. Control animals were treated with distilled water. Each group was monitored daily on weight and lethality for two weeks post-inoculation.

To additionally evaluate the pathogenicity of control and GS-resistant viruses, three groups of mice (10 per group) were infected with a low dose of viruses (5 × 10^2^ TCID_50_ per mouse). After ten days post-inoculation, animals were euthanized, and their lungs were visually studied for post-influenza lesions looking like foci of lung swelling and carnification [63]. The score values of lung pathology were determined as follows: 0: no pathology; 1: 1% to 25% of lung surface is injured; 2: 26% to 50%; 3: 51% to 75%, 4: 76% and more. Lungs were studied by two observers having no information about the virus used in a specific group. Based on the results, the mean pathology score was calculated for each animal group.

#### 3.2.11. Statistical Analysis

Results were represented as means ± standard deviations of the mean. The values of CC50 and IC50’s were calculated using GraphPad PRISM 6.01 software. Animals’ survival curves were analysed by Mantel-Cox test (GraphPad PRISM 6.01 software San Diego, CA, USA), lung pathology scores were compared by Student test (Microsoft Excel).

### 3.3. Computation Details

#### 3.3.1. Protein and Ligand Preparation

To estimation of Ginsamide affinity to HA, geometrical parameters HA-camphecene complex was used for molecular docking procedure. The complex was obtained as a result of a number of molecular modeling procedures described in [43,54]. Amino acide sequence is correspond to PBD code 1RU7 [55]. For calculations, the monomeric form of haemagglutinin was used. The geometric parameters of protein and ligand were restrained optimised in the OPLS4e [64] force field at physiological and low pH values (5.0 ± 0.2).

#### 3.3.2. Bindin Site Analysis

The small hydrophobic pocket located in the place of proteolysis between the long α-helix and two-loop N-terminate of HA_2_ was considered for binding ginsamide. This binding site was selected based on the similarity of the pharmacophore descriptors of camphecene and ginsamide. The binding site is saturated with hydrophobic amino acids such as valine, alanine, tyrosine, phenylalanine, leucine, and isoleucine. V615 and G10 are considered functional amino acids.

For the binding site alignment procedure, we considered the following codes corresponding to various types of HA: H1 (PBD code 1RU7 [55]); H1-pdm (PBD code 3LZG [58]); H3 (pdb code 3EYM [47]); H5 (PDB code 6CF5 [59]) and H7 (PBD code 6ID2 [60]).

#### 3.3.3. Molecular Docking Procedure

Molecular docking procedures and dynamics were performed using Schrodinger Suite (Release 2021-1) software (Schrödinger Release 2021-1: 2021, Schrödinger, LLC., New York, NY, USA). Ginsamide was docked using the forced ligand positioning protocol (IFD) with the following conditions: flexible protein and ligand; grid matrix size of 15 Å; and amino acids (within a radius of 5 Å from the ligand) restrained and optimized, taking into account the influence of the ligand. However, binding energies (ΔG_MM-GBSA_) ligand-protein complexes were estimated using the variable-dielectric generalized Born model, which incorporates residue-dependent effects. The solvent is water.

## 4. Conclusions

In conclusion, we have identified a novel class of cage compounds based on a ginsenol scaffold with high anti-influenza virus activity. The lead compound, ginsamide ((1S,3aR,4R,7aS)-*N*-(2,2,4,7a-tetramethyloctahydro-1,4-etanoinden-3a-yl)-acetamide) demonstrated low cytotoxicity (CC_50_ > 1140 μM) and high virus-inhibiting activity (IC_50_ = 0.152 μM, SI = 7500). This activity was strongly specific for A(H1) viruses, while influenza viruses A of H3, H5, and H7 subtypes, as well as influenza virus B, were non-susceptible to these compounds. Ginsamide appeared to demonstrate the strongest antiviral action when used in the early stages of the viral cycle, its effect being due to the suppression of the fusogenic activity of viral HA. In the animal model of influenza infection, ginsamide protected animals from virus-induced death when administered at high doses (150 mg/kg/day). Lower doses did not result in a substantial decrease in mortality. Ten passages of influenza virus at increasing concentrations of ginsamide resulted in the selection of a resistant strain whose IC_50_ was almost 700 times higher than the initial virus. The development of resistance was associated with three amino acid substitutions in the HA gene that were localized at the receptor-binding, fusion peptide, and proteolytic cleavage sites. Taken together, these results demonstrate the high antiviral potential of ginsenol-based compounds.

## Figures and Tables

**Table 1:** Antiviral activity of the compounds **1a**–**d**, **2**, **3** against influenza virus A/Puerto Rico/8/34 (H1N1) in MDCK cells.

Compound	CC_50_ (µM) ^a^	IC_50_ H1N1 (µM) ^b^	SI ^c^
**1a**	>1140	0.152 ± 0.03	7500
**1b**	128.5 ± 9.4	0.144 ± 0.019	890
**1c**	>1031	0.21 ± 0.029	5000
**1d**	141.3 ± 11.5	9.2 ± 1.1	15
**2**	15.4 ± 1.2	0.45 ± 0.06	34
**3**	>1126	1.13 ± 0.19	1000
Oseltamivir	>200	0.31 ± 0.04	645
Ribavirin	>2000	24.6 ± 3.7	>81
Rimantadine	335 ± 27	67.0 ± 4.9	5

^a^ CC_50_ is the cytotoxic concentration, the concentration resulting in the death of 50% of the cells; ^b^ IC_50_ is the 50% virus-inhibiting concentration, the concentration leading to 50% inhibition of virus replication; ^c^ SI is the selectivity index, the ratio of CC_50_/IC_50_. The data presented are the mean of three independent experiments. The values for CC_50_ and IC_50_ are presented as the mean ± error of the experiment.

**Table 2:** The spectrum of inhibiting the activity of ginsamide against influenza viruses is based on the results of cytoprotection assay.

Virus	CC_50_ (µM) ^a^	IC_50_ H1N1 (µM) ^b^	SI ^c^
A/Puerto Rico/8/1934 (H1N1)	>1140	0.152 ± 0.03	7500
A/California/7/2009 (H1N1) pdm09	>1140	10.65 ± 1.3	107
A/Vladivostok/2/2009 (H1N1)	>1140	0.38 ± 0.051	3000
A/Aichi/2/1968 (H3N2)	>1140	789 ± 82	15
A/Anhui/1/2013 (H7N9)	>1140	125.5 ± 11.8	9
A/Mallard/Pennsylvania/1984 (H5N2)	>1140	133.1 ± 15.6	9
B/Florida/4/2006 (Yamagata-like)	>1140	1125.5 ± 141.4	1

^a^ CC_50_ is the cytotoxic concentration, the concentration resulting in the death of 50% of the cells; ^b^ IC_50_ is the 50% virus-inhibiting concentration, the concentration leading to 50% inhibition of virus replication; ^c^ SI is the selectivity index, the ratio of CC_50_/IC_50_. The data presented are the mean of three independent experiments. The values for CC_50_ and IC_50_ are presented as the mean ± error of the experiment.

## Data Availability

The data presented in this study are available within the article.

## Sample Availability

Samples of the compounds are not available from the authors.

## References

[1] Viasus D., Revuelta J.A.O., Martínez-Montauti J., Carratalà J. (2012). Influenza A(H1N1)pdm09-related pneumonia and other complications. Enferm. Infecc. Microbiol. Clin..

[2] Vousden N., Knight M. (2020). Lessons learned from the A (H1N1) influenza pandemic. Best Pract. Res. Clin. Obstet. Gynaecol..

[3] Karlsson E.A., Marcelin G., Webby R.J., Schultz-Cherry S. (2012). Review on the impact of pregnancy and obesity on influenza virus infection. Influenza Other Respir. Viruses.

[4] Philippon D.A.M., Wu P., Cowling B.J., Lau E.H.Y. (2020). Avian Influenza Human Infections at the Human-Animal Interface. J. Infect. Dis..

[5] Bright R.A., Shay D.K., Shu B., Cox N.J., Klimov A.I. (2006). Adamantane Resistance Among Influenza A Viruses Isolated Early during the 2005–2006 Influenza Season in the United States. JAMA.

[6] Feng E., Ye D., Li J., Zhang D., Wang J., Zhao F., Hilgenfeld R., Zheng M., Jiang H., Liu H. (2012). Recent Advances in Neuraminidase Inhibitor Development as Anti-influenza Drugs. ChemMedChem.

[7] Scott L.J. (2018). Peramivir: A Review in Uncomplicated Influenza. Drugs.

[8] Kashiwagi S., Yoshida S., Yamaguchi H., Niwa S., Mitsui N., Tanigawa M., Shiosakai K., Yamanouchi N., Shiozawa T., Yamaguchi F. (2012). Safety of the long-acting neuraminidase inhibitor laninamivir octanoate hydrate in post-marketing surveillance. Int. J. Antimicrob. Agents.

[9] Hayden F.G., Sugaya N., Hirotsu N., Lee N., de Jong M.D., Hurt A.C., Ishida T., Sekino H., Yamada K., Portsmouth S. (2018). Baloxavir Marboxil for Uncomplicated Influenza in Adults and Adolescents. N. Engl. J. Med..

[10] Bernardini S., Tiezzi A., Masci V.L., Ovidi E. (2018). Natural products for human health: An historical overview of the drug discovery approaches. Nat. Prod. Res..

[11] Salakhutdinov N.F., Volcho K.P., Yarovaya O.I. (2017). Monoterpenes as a renewable source of biologically active compounds. Pure Appl. Chem..

[12] Balunas M.J., Kinghorn A.D. (2005). Drug discovery from medicinal plants. Life Sci..

[13] Rishton G.M. (2008). Natural Products as a Robust Source of New Drugs and Drug Leads: Past Successes and Present Day Issues. Am. J. Cardiol..

[14] Yarovaya O.I., Salakhutdinov N.F. (2021). Mono- and sesquiterpenes as a starting platform for the development of antiviral drugs. Russ. Chem. Rev..

[15] Zielińska-Błajet M., Feder-Kubis J. (2020). Monoterpenes and their derivatives—Recent development in biological and medical applications. Int. J. Mol. Sci..

[16] Chen Z., Cui Q., Caffrey M., Rong L., Du R. (2021). Small molecule inhibitors of influenza virus entry. Pharmaceuticals.

[17] Sokolova A.S., Yarovaya O.I., Baranova D.V., Galochkina A.V., Shtro A.A., Kireeva M.V., Borisevich S.S., Gatilov Y.V., Zarubaev V.V., Salakhutdinov N.F. (2021). Quaternary ammonium salts based on (−)-borneol as effective inhibitors of influenza virus. Arch. Virol..

[18] Sokolova A.S., Yarovaya O.I., Baev D.S., Shernyukov A.V., Shtro A.A., Zarubaev V.V., Salakhutdinov N.F. (2017). Aliphatic and alicyclic camphor imines as effective inhibitors of influenza virus H1N1. Eur. J. Med. Chem..

[19] Zarubaev V.V., Garshinina A.V., Tretiak T.S., Fedorova V.A., Shtro A.A., Sokolova A.S., Yarovaya O.I., Salakhutdinov N.F. (2015). Broad range of inhibiting action of novel camphor-based compound with anti-hemagglutinin activity against influenza viruses in vitro and in vivo. Antivir. Res..

[20] Sokolova A.S., Yarovaya O.I., Zybkina A.V., Mordvinova E.D., Shcherbakova N.S., Zaykovskaya A.V., Baev D.S., Tolstikova T.G., Shcherbakov D.N., Pyankov O.V. (2020). Monoterpenoid-based inhibitors of filoviruses targeting the glycoprotein-mediated entry process. Eur. J. Med. Chem..

[21] Sokolova A.S., Putilova V.P., Yarovaya O.I., Zybkina A.V., Mordvinova E.D., Zaykovskaya A.V., Shcherbakov D.N., Orshanskaya I.R., Sinegubova E.O., Esaulkova I.L. (2021). Synthesis and Antiviral Activity of Camphene Derivatives against Different Types of Viruses. Molecules.

[22] Sokolova A.S., Kovaleva K.S., Yarovaya O.I., Bormotov N.I., Shishkina L.N., Serova O.A., Sergeev A.A., Agafonov A.P., Maksuytov R.A., Salakhutdinov N.F. (2021). (+)-Camphor and (−)-borneol derivatives as potential anti-orthopoxvirus agents. Arch. Pharm..

[23] Yarovaya O.I., Kovaleva K.S., Zaykovskaya A.A., Yashina L.N., Scherbakova N.S., Scherbakov D.N., Borisevich S.S., Zubkov F.I., Antonova A.S., Peshkov R.Y. (2021). New class of hantaan virus inhibitors based on conjugation of the isoindole fragment to (+)-camphor or (−)-fenchone hydrazonesv. Bioorg. Med. Chem. Lett..

[24] Astani A., Reichling J., Schnitzler P. (2011). Screening for Antiviral Activities of Isolated Compounds from Essential Oils. Evid. Based Complement. Altern. Med..

[25] Hu G., Peng C., Xie X., Zhang S., Cao X. (2017). Availability, Pharmaceutics, Security, Pharmacokinetics, and Pharmacological Activities of Patchouli Alcohol. Evid. Based Complement. Altern. Med..

[26] Wu H., Li B., Wang X., Jin M., Wang G. (2011). Inhibitory Effect and Possible Mechanism of Action of Patchouli Alcohol against Influenza A (H2N2) Virus. Molecules.

[27] Zheng G.-Q., Kenney P.M., Lam L.K.T. (1992). Sesquiterpenes from Clove (*Eugenia caryophyllata*) as Potential Anticarcinogenic Agents. J. Nat. Prod..

[28] Sylvestre M., Legault J., Dufour D., Pichette A. (2005). Chemical composition and anticancer activity of leaf essential oil of *Myrica gale* L. Phytomedicine.

[29] Sylvestre M., Pichette A., Lavoie S., Longtin A., Legault J. (2007). Composition and cytotoxic activity of the leaf essential oil of *Comptonia peregrina* (L.) Coulter. Phytother. Res..

[30] Goren A.C., Piozzi F., Akcicek E., Kılıç T., Çarıkçı S., Mozioğlu E., Setzer W.N. (2011). Essential oil composition of twenty-two Stachys species (mountain tea) and their biological activities. Phytochem. Lett..

[31] Sharma C., Sadek B., Goyal S.N., Sinha S., Kamal M.A., Ojha S. (2015). Small Molecules from Nature Targeting G-Protein Coupled Cannabinoid Receptors: Potential Leads for Drug Discovery and Development. Evid. Based Complement. Altern. Med..

[32] Ghelardini C., Galeotti N., Mannelli L.D.C., Mazzanti G., Bartolini A. (2001). Local anaesthetic activity of β-caryophyllene. Il Farm..

[33] Fidyt K., Fiedorowicz A., Strządała L., Szumny A. (2016). β-caryophyllene and β-caryophyllene oxide-natural compounds of anticancer and analgesic properties. Cancer Med..

[34] Legault J., Pichette A. (2010). Potentiating effect of β-caryophyllene on anticancer activity of α-humulene, isocaryophyllene and paclitaxel. J. Pharm. Pharmacol..

[35] Neta M.C.S., Vittorazzi C., Guimarães A.C., Martins J.D.L., Fronza M., Endringer D.C., Scherer R. (2017). Effects of β-caryophyllene and Murraya paniculata essential oil in the murine hepatoma cells and in the bacteria and fungi 24-h time–kill curve studies. Pharm. Biol..

[36] Fitjer L., Malich A., Paschke C., Kluge S., Gerke R., Rissom B., Weiser J., Noltemeyer M. (1995). Rearrangement of (-)-β-Caryophyllene. A Product Analysis and Force Field Study. J. Am. Chem. Soc..

[37] Yarovaya O.I., Korchagina D.V., Rybalova T.V., Gatilov Y.V., Polovinka M.P., Barkhash V.A. (2004). Reactions of caryophyllene, isocaryophyllene, and their epoxy derivatives with acetonitrile under Ritter reaction conditions. Russ. J. Org. Chem..

[38] Chen M.-E., Chen X.-W., Hu Y.-H., Ye R., Lv J.-W., Li B., Zhang F.-M. (2021). Recent advances of Ritter reaction and its synthetic applications. Org. Chem. Front..

[39] Ziarani G.M., Hasankiadeh F.S., Mohajer F. (2020). Recent Applications of Ritter Reactions in Organic Syntheses. ChemistrySelect.

[40] Bolsakova J., Jirgensons A. (2017). The Ritter reaction for the synthesis of heterocycles. Chem. Heterocycl. Compd..

[41] Cho I.H. (2015). Volatile compounds of ginseng (*Panax* sp.): A review. J. Korean Soc. Appl. Biol. Chem..

[42] Salomatina O.V., Yarovaya O.I., Korchagina D.V., Gatilov Y.V., Polovinka M.P., Barkhash V.A. (2005). Transformations of isocaryophyllene diepoxide under conditions of homogeneous and heterogeneous acid catalysis. Russ. J. Org. Chem..

[43] Zarubaev V.V., Pushkina E.A., Borisevich S.S., Galochkina A.V., Garshinina A.V., Shtro A.A., Egorova A.A., Sokolova A.S., Khursan S.L., Yarovaya O.I. (2018). Selection of influenza virus resistant to the novel camphor-based antiviral camphecene results in loss of pathogenicity. Virology.

[44] Scholtissek C., Quack G., Klenk H., Webster R. (1998). How to overcome resistance of influenza A viruses against adamantane derivatives. Antivir. Res..

[45] Hoopes J.D., Driebe E.M., Kelley E., Engelthaler D.M., Keim P.S., Perelson A.S., Rong L., Went G.T., Nguyen J.T. (2011). Triple Combination Antiviral Drug (TCAD) Composed of Amantadine, Oseltamivir, and Ribavirin Impedes the Selection of Drug-Resistant Influenza A Virus. PLoS ONE.

[46] Nakazawa M., Kadowaki S., Watanabe I., Kadowaki Y., Takei M., Fukuda H. (2008). PA subunit of RNA polymerase as a promising target for anti-influenza virus agents. Antivir. Res..

[47] Russell R.J., Kerry P.S., Stevens D.J., Steinhauer D.A., Martin S.R., Gamblin S.J., Skehel J.J. (2008). Structure of influenza hemagglutinin in complex with an inhibitor of membrane fusion. Proc. Natl. Acad. Sci. USA.

[48] Motohashi Y., Igarashi M., Okamatsu M., Noshi T., Sakoda Y., Yamamoto N., Ito K., Yoshida R., Kida H. (2013). Antiviral activity of stachyflin on influenza A viruses of different hemagglutinin subtypes. Virol. J..

[49] Yanagita H., Yamamoto N., Fuji H., Liu X., Ogata M., Yokota M., Takaku H., Hasegawa H., Odagiri T., Tashiro M. (2012). Mechanism of drug resistance of hemagglutinin of influenza virus and potent scaffolds inhibiting its function. ACS Chem. Biol..

[50] Tang G., Lin X., Qiu Z., Li W., Zhu L., Wang L., Li S., Li H., Lin W., Yang M. (2011). Design and synthesis of benzenesulfonamide derivatives as potent anti-influenza hemagglutinin inhibitors. ACS Med. Chem. Lett..

[51] Combrink K.D., Gulgeze H.B., Yu K.L., Pearce B.C., Trehan A.K., Wei J., Deshpande M., Krystal M., Torri A., Luo G. (2000). Salicylamide inhibitors of influenza virus fusion. Bioorg. Med. Chem. Lett..

[52] Plotch S.J., O’Hara B., Morin J., Palant O., LaRocque J., Bloom J.D., Lang S.A., DiGrandi M.J., Bradley M., Nilakantan R. (1999). Inhibition of Influenza A Virus Replication by Compounds Interfering with the Fusogenic Function of the Viral Hemagglutinin. J. Virol..

[53] Li J., Liu B., Chang G., Hu Y., Zhan D., Xia Y., Li Y., Yang Y., Zhu Q. (2011). Virulence of H5N1 virus in mice attenuates after in vitro serial passages. Virol. J..

[54] Borisevich S.S., Gureev M.A., Yarovaya O.I., Zarubaev V.V., Kostin G.A., Porozov Y.B., Salakhutdinov N.F. (2021). Can molecular dynamics explain decreased pathogenicity in mutant camphecene-resistant influenza virus?. J. Biomol. Struct. Dyn..

[55] Gamblin S.J., Haire L.F., Russell R.J., Stevens D.J., Xiao B., Ha Y., Vasisht N., Steinhauer D.A., Daniels R.S., Elliot A. (2004). The structure and receptor binding properties of the 1918 influenza hemagglutinin. Science.

[56] Berman H.M., Westbrook J., Feng Z., Gilliland G., Bhat T.N., Weissig H., Shindyalov I.N., Bourne P.E. (2000). The Protein Data Bank. Nucleic Acids Res..

[57] Sokolova A.S., Yarovaya O.I., Shernyukov A.V., Gatilov Y.V., Razumova Y.V., Zarubaev V.V., Tretiak T.S., Pokrovsky A.G., Kiselev O.I., Salakhutdinov N.F. (2015). Discovery of a new class of antiviral compounds: Camphor imine derivatives. Eur. J. Med. Chem..

[58] Xu R., Ekiert D.C., Krause J.C., Hai R., Crowe J.E., Wilson I.A. (2010). Structural Basis of Preexisting Immunity to the 2009 H1N1 Pandemic Influenza Virus. Science.

[59] Kadam R.U., Wilson I.A. (2018). A small-molecule fragment that emulates binding of receptor and broadly neutralizing antibodies to influenza A hemagglutinin. Proc. Natl. Acad. Sci. USA.

[60] Xu Y., Peng R., Zhang W., Qi J., Song H., Liu S., Wang H., Wang M., Xiao H., Fu L. (2019). Avian-to-Human Receptor-Binding Adaptation of Avian H7N9 Influenza Virus Hemagglutinin. Cell Rep..

[61] Mosmann T. (1983). Rapid colorimetric assay for cellular growth and survival: Application to proliferation and cytotoxicity assays. J. Immunol. Methods.

[62] Maeda T., Ohnishi S. (1980). Activation of influenza virus by acidic media causes hemolysis and fusion of erythrocytes. FEBS Lett..

[63] Kumar P.A., Hu Y., Yamamoto Y., Hoe N.B., Wei T.S., Mu D., Sun Y., Joo L.S., Dagher R., Zielonka E.M. (2011). Distal Airway Stem Cells Yield Alveoli In Vitro and during Lung Regeneration following H1N1 Influenza Infection. Cell.

[64] Lu C., Wu C., Ghoreishi D., Chen W., Wang L., Damm W., Ross G.A., Dahlgren M.K., Russell E., Von Bargen C.D. (2021). OPLS4: Improving Force Field Accuracy on Challenging Regimes of Chemical Space. J. Chem. Theory Comput..

